# Effector prediction in host-pathogen interaction based on a Markov model of a ubiquitous EPIYA motif

**DOI:** 10.1186/1471-2164-11-S3-S1

**Published:** 2010-12-01

**Authors:** Shunfu Xu, Chao Zhang, Yi Miao, Jianjiong Gao, Dong Xu

**Affiliations:** 1 Department of Gastroenterology, the First Affiliated Hospital of Nanjing Medical University, Jiangsu 210029, China; 2 Department of Computer Science, University of Missouri, Columbia, MO 65211, USA; 3 C.S. Bond Life Sciences Center, University of Missouri, Columbia, MO 65211, USA; 4 Department of General Surgery, the First Affiliated Hospital of Nanjing Medical University, Jiangsu 210029, China

## Abstract

**Background:**

Effector secretion is a common strategy of pathogen in mediating host-pathogen interaction. Eight EPIYA-motif containing effectors have recently been discovered in six pathogens. Once these effectors enter host cells through type III/IV secretion systems (T3SS/T4SS), tyrosine in the EPIYA motif is phosphorylated, which triggers effectors binding other proteins to manipulate host-cell functions. The objectives of this study are to evaluate the distribution pattern of EPIYA motif in broad biological species, to predict potential effectors with EPIYA motif, and to suggest roles and biological functions of potential effectors in host-pathogen interactions.

**Results:**

A hidden Markov model (HMM) of five amino acids was built for the EPIYA-motif based on the eight known effectors. Using this HMM to search the non-redundant protein database containing 9,216,047 sequences, we obtained 107,231 sequences with at least one EPIYA motif occurrence and 3115 sequences with multiple repeats of the EPIYA motif. Although the EPIYA motif exists among broad species, it is significantly over-represented in some particular groups of species. For those proteins containing at least four copies of EPIYA motif, most of them are from intracellular bacteria, extracellular bacteria with T3SS or T4SS or intracellular protozoan parasites. By combining the EPIYA motif and the adjacent SH2 binding motifs (KK, R4, Tarp and Tir), we built HMMs of nine amino acids and predicted many potential effectors in bacteria and protista by the HMMs. Some potential effectors for pathogens (such as *Lawsonia intracellularis, Plasmodium falciparum* and *Leishmania major*) are suggested.

**Conclusions:**

Our study indicates that the EPIYA motif may be a ubiquitous functional site for effectors that play an important pathogenicity role in mediating host-pathogen interactions. We suggest that some intracellular protozoan parasites could secrete EPIYA-motif containing effectors through secretion systems similar to the T3SS/T4SS in bacteria. Our predicted effectors provide useful hypotheses for further studies.

## Background

As a complex and interesting relation between organisms in ecology and evolution, host-pathogen interaction is a basis of infectious diseases [1] . Pathogens span a broad spectrum of biological species, including viruses, bacteria, fungi, protozoa, and multicellular parasites. In all these cases, a pathogen causing an infection usually exhibits an extensive interaction with the host during pathogenesis. The cross-talks between a host and a pathogen allow the pathogen to successfully invade the host organism, to breach its immune defence, as well as to replicate and persist within the organism. One of the most important and therefore widely studied groups of host- pathogen interactions is the interaction between pathogen protein (effector) and host cells. Effectors are secreted from pathogens' secretion systems. So far five types of secretion systems have been identified (Types I-V). Among them, T3SS (Type III Secretion System) and T4SS (Type IV Secretion System) can cross bacterial cell walls and host eukaryotic cell membranes to deliver effectors into host cells directly without going through extracellular matrix [2] . Those effectors can manipulate host cell functions once entering host cell [2] . Identifying effectors and exploring their molecular mechanisms not only are critical to understanding the disease mechanisms but also provide theoretical foundations for infectious disease diagnosis, prognosis and treatment [3,4] .

A well-studied effector is Cytotoxin-associated gene A (CagA), a most important virulence factor in *Helicobacter pylori* (*H. pylori*), which is one of the major pathogens of upper gastrointestinal diseases (e.g., peptic ulcer and gastric cancer) [5] . CagA can be delivered into gastric epithelial cells by the T4SS of *H. pylori.* Recent studies of CagA sequences found that they have a variable region within which the EPIYA (glutamic acid-proline-isoleucine-tyrosine-alanine) motif repeats from once to seven times. Tyrosine in the EPIYA motif can be phosphorylated in the host cell. The phosphorylated CagA protein binds to a phosphatase SHP-2, which will interfere with the signal transduction pathway of the host cell and manipulate cell growth, differentiation and apoptosis [6-8] . This interference causes a restructure of the host cell cytoskeleton, cell scattering as well as invasive growth of cells, and formation of hummingbird phenotype with gastric epithelial cells. Such a process not only is considered an important strategy of interaction between *H. pylori* and host cell, but also is the most significant mechanism of pathogenesis and carcinogenesis of *H.pylori*[9-11] .

In recent years, studies have discovered other pathogens that can also secrete effectors to manipulate the host cells through phosphorylation during the interaction process between hosts and pathogens (e.g. *Anaplasma phagocytophilum*[12,13] and *Bartonella henselae*[14-16] ). These effectors cause rearrangements of host cell cytoskeleton, NF-kB activation and apoptosis inhibition [17] . Table 1 lists eight effectors from six pathogens. They contain 28 experimentally identified phosphorylation sequences, all of which have the similar pattern to the EPIYA motif in CagA [15] . This finding leads to our hypothesis that the EPIYA-like motif and its phosphorylation, together with its interference of host cells, may be a general mechanism of pathogenesis. Based on this novel hypothesis, we used the effectors in Table 1 to build an EPIYA-motif-based hidden Markov model (HMM), and then searched the current protein database to identify more proteins with the EPIYA motif. Through studying the distribution and features of EPIYA motif in different species and genuses, we attempted to better understand the function of EPIYA motif, especially the role of EPIYA motif during the interaction process between pathogens and hosts.

**Table 1:** Experimentally determined tyrosine-phosphorylated effectors and their motifs

Effector	Pathogen	Locus of protein	Motif (phosphorylated Y position)					
CagA	*H.Ppylori*	NP_207343	EPIYAKVNK	Y-899	EPIYTQVAK	Y-918	EPIYATIDD	Y-972

Ankyrin	*Anaplasma phagocytophilum*	ABB84853	ESIYEEIKD	Y-940	ESIYEEIKD	Y-967	ESIYEEIKD	Y-994
			EDLYATVGA	Y-1028	ESIYADPFD	Y-1056	ESIYADPFA	Y-1074
			EPIYATVKK	Y-1098				

BepD	*Bartonella henselae*	YP_034066	EPLYAQVNK	Y-32	NPLYEGVGG	Y-114	NPLYEGVGS	Y-176
			EPLYAQVNK	Y-211	NPLYEGVGG	Y-293	NPLYEGVGP	Y-355

BepE	*Bartonella henselae*	YP_034067	EPLYATVNK	Y-37	ETIYTTVSS	Y-91

BepF	*Bartonella henselae*	YP_034068	TPLYATPSP	Y-149	EPLYATPLP	Y-213	EPLYATPLP	Y-241
			EPLYATAAP	Y-297	EPLYATPLP	Y-269

Tir	*Escherichia Eoli*	AAC38390	EHIYDEVAA	Y-474

Tir	*Citrobacter rodentium*	AAL06376	EPIYDEVAP	Y-468

Trap	*Chlamydie trachomatis*	YP_001654788	ENIYENIYE	Y-136	ENIYENIYE	Y-238	ENIYENIYE	Y-390

CagA: cytotoxin associated gene A [7,8,10,52-56]; BepD: *Bartonella henselae* protein D [14-16,57]; BepE: *Bartonella henselae* protein E [14-16,57]; BepF: *Bartonella henselae* protein F [14-16,57]; Tir: translocated intimin receptor [58-60] ; Trap: Translocated actin-recruiting protein [61-63] . The first five amino acids of the listed sequences in the table correspond to the EPIYA motif.

## Results & discussion

### 1. Building and using hidden Markov model

**Figure 1:**
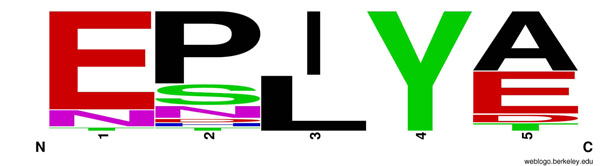
Sequence logo of the known EPIYA motif sequences

Using the 28 experimentally identified phosphorylated motif sequences in Table 1, we built the sequence logo as shown in Figure 1. In this logo, the fourth position of the EPIYA motif is always tyrosine (Y), which can be phosphorylated. The first and third positions have small variations. The amino acids in the first position are primarily glutamic acid (E), together with asparagine (N). Most residues in the third position are isoleucine (I) and leucine (L), two very similar amino acids. The second and fifth positions have big variations. The second position varies from proline (P), serine (S) to asparagine (N). The fifth position mainly contains alanine (A), glutamic acid (E) and aspartic acid (D). These 28 sequences were used to build and calibrate the HMM by applying Hmmer 2.3.2 (http://hmmer.janelia.org). We then employed the HMM to search the protein non-redundant (NR) database, which contains 9,216,047 protein sequences. The search yielded 107,231 sequences containing at least one copy of EPIYA motif and 3115 sequences with multiple repeats of the EPIYA motif, where the highest number of repeats in a single protein is 29 (see Table 2).

**Table 2:** Distribution of protein sequences containing the EPIYA motif

Number of motif repeats in one protein	Number of protein sequences	Observed Frequency	Expected Frequency
29	1	1.09E-07	3.44E-57

14	2	2.17E-07	5.52E-28

13	2	2.17E-07	4.88E-26

12	1	1.09E-07	4.32E-24

10	1	1.09E-07	3.39E-20

9	3	3.26E-07	3.00E-18

8	6	6.51E-07	2.65E-16

7	10	1.09E-06	2.35E-14

6	32	3.47E-06	2.08E-12

5	55	5.97E-06	1.84E-10

4	173	1.88E-05	1.63E-08

3	916	9.94E-05	1.44E-06

2	1913	2.08E-04	1.28E-04

1	104116	1.13E-02	1.13E-02

Expected frequency is the expected probability if the combination of the motif in a protein sequence is random.

We found that the repeats of EPIYA motif in a protein are highly non-random. As the probability of one protein sequence having a copy of EPIYA motif is 1.13E-02 (104,116/9,216,047), the expected probabilities of one protein sequence containing 24 copies of EPIYA motif are (1.13E-02)^2^=1.28E-04, (1.13E-02)^3^=1.44E-06, and (1.13E-02)^4^=1.63E-08, respectively, assuming the combination of the motif in a sequence is random. The observed probabilities of one sequence containing multiple copies of EPIYA motif are much larger than the expected probabilities as shown in Table 2. Hence, the repeats of the EPIYA motif may have been resulted from evolution with biological significance. This is also reflected in Table 1, where most effectors with known EPIYA motif have 2-7 motif repeats. Thus, we suggest that multiple copies of EPIYA motif in the same protein are more likely to be functional than single motif occurrence.

### 2. Distribution pattern of EPIYA motif among species

The NR database contains proteins sequences from 27,432 genuses and 121,718 species. Among them the sequences from 2675 genuses and 4646 species contain at least one copy of EPIYA motif, and 368 genuses and 587 species have proteins containing at least two copies of EPIYA motif. The proteins with the EPIYA motif are mainly distributed in lower organisms. As shown in Table 3, the probability of a genus/species containing proteins with the EPIYA motif in archaea, viruses or bacteria is much higher than that in eukaryotes. This indicates that with evolution advanced species mostly lost the EPIYA motif together with its functions for host- pathogen interactions.

**Table 3:** Distribution of EPIYA-motif containing proteins at genus and species levels (as of July 6^th^ 2009)

Groups	Number of genuses					Number of species				
	**total**	**With copies of motif≥1 (%)**		**With copies of motif≥2 (%)**		**total**	**With copies of motif≥1 (%)**		**With copies of motif≥2 (%)**	

Archaea	109	49	44.95%	19	17.43%	330	90	27.27%	28	8.48%

Viruses	623	221	35.47%	18	2.89%	6443	433	6.72%	30	0.47%

Bacteria	1198	560	46.74%	209	17.45%	6291	1398	22.22%	360	5.72%

Eukaryota	35499	1828	5.15%	122	0.34%	108654	2725	2.51%	169	0.16%

-Protista	1263	109	8.63%	18	1.43%	3747	186	4.96%	32	0.85%

-Fungi	1509	121	8.02%	40	2.65%	5772	206	3.57%	52	0.90%

-Metazoa	22309	662	2.97%	49	0.22%	62097	826	1.33%	68	0.11%

-Viridiplantae	10418	936	8.98%	15	0.14%	37038	1507	4.07%	17	0.05%

total	37429	2658	7.10%	368	0.98%	121718	4646	3.82%	587	0.48%

Data in this table presents the numbers of genuses/species with proteins containing the EPIYA motif versus total number of genuses/species in NR. The Eukaryota group is divided into protista, fungi, metzoa and viridiplantae.

We listed top 10 species and genuses with most EPIYA-motif containing proteins for the groups of archaea, viruses, bacteria, protista, fungi, metazoa and viridiplantae (Additional File 1). In archaea, *Methanococcus* is the genus that includes the most EPIYA-motif containing proteins. In viruses, *Potyvirus* is the highest in number of EPIYA-motif containing proteins among genuses while *Bovine Viral Diarrhea Virus* is the highest among species. The top four genuses (and the corresponding species) in bacteria are *Helicobacter* (*Helicobacter pylori*)*, Clostridum* (*Clostridum botulinum, Clostridum perfringens*)*, Bacillus* (*Bacillus cereus*) and *Anaplasma* (*Anaplasma phagocytophilum*)*. Plasmodium* (*Plasmodium falciparum*) and *Tetrahymena* (*Tetrahymena thermophila*) are the top genuses in protista. In fungi and viridiplantae, the corresponding top genuses are *Candida* (*Candida tropicalis*) and *Oryza* (*Oryza sativa*)*,* respectively. Two well-studied genuses *Drosophila* (*Drosophila melanogaster*) and *Homo* (*Homo sapiens*) take the top two in metazoa. It should be noted that the data in Additional File 1 are biased, with widely studied species such as *Helicobacter pylori* having the same gene sequenced many times, while some other species have incomplete proteomes. Nevertheless, this table in Additional File 1 provides some interesting reference for known and putative pathogens with effectors.

**Figure 2:**
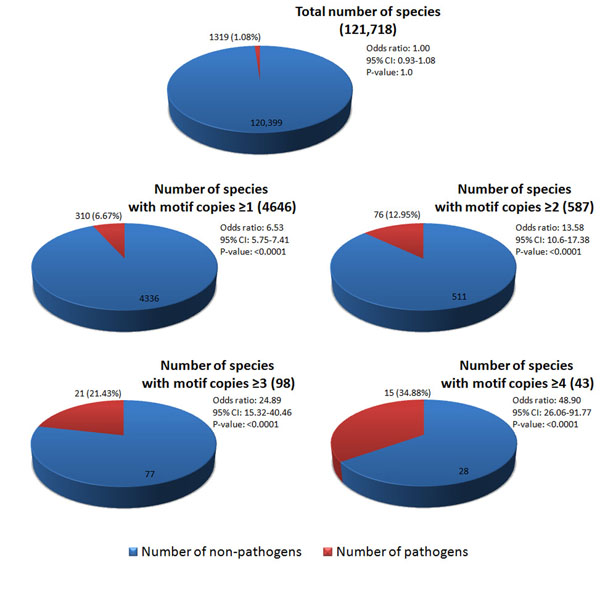
**Relationship between number of EPIYA motif copies and number of species in known pathogens** The p-values are calculated from chi-square test statistic between the group and the control (the total with all the species as the background). The list of pathogens is shown in Table 4

Bacterial pathogens can be divided to two types. Some can enter host cells, e.g., *Chlamydia* and *Anaplasma.* They are known as intracellular pathogens and most of them have T3SS or T4SS. Some other bacteria are extracellular pathogens with T3SS or T4SS, such as *Helicobacter pylori* and *Campylobacter.* As shown in Additional File 1, numerous bacteria containing multiple copies of EPIYA motif belong to pathogens, such as *Anaplasma (Anaplasma phagocytophilumn,* ranking 4th in the species list) and *Chlamydia* (*Chlamydia trachomatis,* ranking 10th in the species list), both of which are intracellular pathogens. *Helicobacter* (ranking 1st in both genus and species list) and *Campylobacter* (ranking 10th in the genus list) contain T4SS [18] . Some other T4SS-containing species are not listed in Additional File 1, such as *Wolbachia* (ranking 13th in the genus list), *Escherichia* (ranking 18th in the genus list), *Mycobacterium* (ranking 23rd in the genus list) and *Bartonella* (ranking 27th in the genus list). Furthermore, we also found that in protista most top 10 species are unicellular parasites that can live in host cells to survive and reproduce by subverting of signalling pathways and inhibiting apoptosis of host cells [19] . However, the pathogens mediators responsible for this modulation are still unknown [20] . Those intracellular protozoan parasites include *Plasmodium* (ranking 1st in the genus list), *Leishmania, Trichomonas, Cryptosporidium* and *Giardia* (correspondingly ranking 58th in the genus list). Table 4 lists most known pathogens including intracellular bacteria (*Mycobacteriaceae, Legionellales, Chlamydiales, Rickettsiales* and *Listeriaceae*)*,* extracellular bacteria with T3SS or T4SS (*Enterobacteriaceae, Campylobacterales* and *Rhizobiales*) and intracellular protozoan parasites (*Apicomplexa* and *Kinetoplastida*)*,* representing 1319 species in total with and without the EPIYA motif. We analyzed the distribution of EPIYA motif in the potential effectors in these pathogens in Figure 2. 310 out of 4646 species with the EPYIA motif belong to such pathogens, and the percentage (310/4646=6.67%) is much higher than that of such pathogens (with and without the EPIYA motif) in all species (1319/121,718=1.08%) (p-value<0.0001, odds ratio=6.54). We also found that the percentage of species with the EPYIA motif belonging to pathogens increases significantly with the increase of EPIYA motif repeats in a protein sequence.

**Table 4:** Known intracellular bacterial pathogens or bacteria containing III/IV type secretion system, and intracellular parasitic protozoan

Bacteria			Protista		
**Genus**	Type	Number of species	**Genus**	Type	Number of species

**Enterobacteriaceae**		245	**Apicomplexa**		187

*Salmonella*	T3SS		*Babesia*	IPP	

*Yersinia pestis*	T3SS		*Cryptosporidium*	IPP	

*Shigella*	T3SS		*Plasmodium*	IPP	

*Escherichia*	T3SS		*Isospora*	IPP	

**Campylobacterales**		76	*Toxoplasma*	IPP	

*Campylobacter*	T4SS		*Theileria*	IPP	

*Helicobacter*	T4SS				

*Wolinella*	T4SS				

**Rhizobiales**		346	**Kinetoplastida**		120

Brucella	IPB		*Leishmania*	IPP	

*Bartonella*	IPB		*Trypanosoma*	IPP	

*Agrobacterium*	T4SS				

**Rickettsiales**		83			

*Anaplasma*	IPB				

*Ehrlichia*	IPB				

*Wolbachia*	IPB				

*Rickettsia*	IPB				

Chlamydiae		23			

*Chlamydia*	IPB				

**Legionellales**		62			

*Legionella*	IPB				

*Coxiella*	IPB				

*Rickettsiella*	IPB				

**Mycobacteriaceae**		168			

*Mycobacterium*	IPB				

**Listeriaceae**		9			

*Listeria*	IPB				

IPB: intracellular parasitic bacteria; IPP: intracellular parasitic protozoan; T3SS: type III secretion system; T4SS: type IV secretion system.

### 3. Distribution pattern of EPIYA-motif containing proteins

In our search result, there are totally 3115 protein sequences with at least two EPIYA motif repeats. Among them, most are CagA of *Helicobacter pylori* as this protein has been extensively studied, and 689 out of 3115 are hypothetical proteins whose functions have not been identified. Based on protein functions, the top 40 proteins ranking by occurrence under each protein function type are widely distributed. They not only exist in achaea, viruses and bacteria, but also are found in protista, metazoa and viridiplantae. Besides the known EPIYA-motif containing effectors ankryin and Tarp, they also involve enzymes related to DNA, ATP and tRNA, transcription regulators, tumor suppressors, different types of kinases, zinc-finger proteins, ubiquitin and various metabolic enzymes (Table 5).

**Table 5:** Distribution of top 40 protein sequences containing at least two copies of EPIYA motif

Protein Name	Number of proteins (number of genuses)							
	**Total**	**Archaea**	**Viruses**	**Bacteria**	**Protista**	**Fungi**	**Metazoa**	**Viridiplantae**

CagA	1015(1)			1015(1)				

hypothetical protein	689(186)	15(10)	10(6)	242(88)	162(19)	78(28)	127(25)	55(11)

ATP*	81(21)	2(2)		68(11)	6(4)	4(3)		1(1)

Ankryin	55(7)			51(3)			4(4)	

DNA*	52(34)	3(3)		40(25)	7(4)	1(1)		1(1)

Kinase	43(28)	5(2)		23(15)	4(3)		11(8)	

zinc finger protein	43(11)						43(11)	

TPR repeat protein	33(15)			33(15)				

Polyprotein	24(2)		24(2)					

SecA	23(14)			23(14)				

Peptidase	19(12)	1(1)		16(9)	1(1)		1(1)	

dynein heavy chain	17(13)				4(2)	1(1)	11(9)	1(1)

elongation factor 2	15(7)				10(2)	1(1)	4(4)	

Palmdelphin	14(9)						14(9)	

tRNA*	14(11)	2(1)		10(8)	1(1)	1(1)		

glycogen synthase	13(1)			13(1)				

GTP-binding	13(3)			12(2)	1(1)			

transcriptional regulator	13(8)	1(1)		9(4)		3(3)		

unc-119 homolog	13(6)						13(6)	

FAT tumor suppressor homolog 3	12(9)						12(9)	

nuclear ribonucleoprotein	12(9)				1(1)	10(7)		1(1)

4-alpha-glucanotransferase	9(1)			9(1)				

paternally expressed 3	8(6)						8(6)	

Striatin	8(7)						8(7)	

Tarp	8(1)			8(1)				

nuclear autoantigen	7(6)						7(6)	

putative mannosyltransferase	7(1)			7(1)				

Ubiquitin	7(6)				5(4)	2(2)		

26S proteasome regulatory subunit	6(3)							6(3)

cell division protein	6(4)	3(3)		1(1)				

centaurin, delta 3	6(5)						6(5)	

fat tumor suppressor homolog 2	6(5)						6(5)	

glycosyl transferase	6(5)			6(5)				

guanine nucleotide exchange factor	6(6)						6(6)	

cytochrome c oxidase subunit VI	5(4)				5(4)			

PEG3	5(5)						5(5)	

polyketide synthase	5(4)			3(3)	2(1)			

polysaccharide biosynthesis protein	5(3)			5(3)				

TatD-related deoxyribonuclease	5(1)			5(1)				

translation initiation factor	5(4)			2(2)	2(1)	1(1)		

ATP* includes ATPase, ABC transporter, ATP-binding protein, and ATP-dependent helicase; DNA* includes DNA photolyase, DNA primase, DNA repair protein, DNA-binding protein, and DNA mismatch repair protein; kinases* includes histidine kinase, protein kinase, hexokinase, serine kinase, and fyn-related kinase; tRNA* includes tRNA synthetase, tRNA formyltransferase, and tRNA ligase.

Although many of these predicted effectors are false positives and the EPIYA motif may not be functional in them, a significant portion of them is likely to be true effectors. As known effectors, ankyrin and TPR (tetratricopeptide repeat) are related to protein-protein interaction [21,22] . Considering the sequence similarity of the above proteins, 44 sequences of ankryin are highly similar among each other and come exclusively from *Anaplasma phagocytophilum, Wolbachia endosymbiont* and *Ehrlichia sp.,* all of which belong to *Rickettsiales.* Except the sequences from *Haliangium ochraceum* (ZP_03879805 and ZP_03880192), other sequences of TPR repeat-containing proteins are also similar and they are from *Trichodesmium erythraeum, Stigmatella aurantiaca, Acaryochloris marina, Cyanothece sp.* and *Microcoleus chthonoplastese.* For the 20 hypothetical proteins, YP_034066 and YP_001610012 (*Bartonella*), YP_153762 and YP_002563468 (*Anaplasma*), XP_001623017 and XP_001636029 (*Nematostella*), ZP_01620341 and ZP_01622571 (*Lyngbya*), XP_001468598 and XP_001686356 (*Leishmania*) are similar pairs in sequences (with more than 30% sequence identity in each pair), and two proteins in a pair are from the same genus. The EPIYA motif in these proteins is highly conserved during evolution, and it may play similar roles as the motif in CagA.

Among proteins containing at least four copies of EPIYA motif (Additional File 2) with 286 sequences in total, most of them are from bacteria, especially from intracellular bacterial pathogens or extracellular bacterial pathogens with T3SS or T4SS, and some are from protist, e.g., intracellular protozoan parasites. Four out of eight known effectors (CagA, Ankyrin, BepD, and Tarp) are found in these sequences, and thus other proteins from bacteria and protista in Additional File 2 may also be effectors. An interesting observation is that the percentage of protein sequences having the EPIYA motif in archaea is the highest among all groups (see Table 3), but none of these archaeal proteins contain four or more copies of EPIYA motif. Previous studies revealed that CagA sequences with more EPIYA-motif occurrences are more virulent [6] . Since archaea and other organisms have relationships of either mutualism or commensalsim and till now there is no clear evidence for the existence of archea parasites [23,24] , it is unlikely that the archaeal proteins containing the EPIYA motif act as pathogen effectors. Compared to other groups, archaea is not well studied, but we can still find some interesting examples, such as *Methanobrevibacter smithii* (ranking 5th in the species list in Additional File 1). It is the most common commensal archaea in the human gut and plays an important role in digesting polysaccharides, while it may not benefit the host directly. We speculate that EPIYA- motif containing proteins of *Methanobrevibacter smithii* may have some biological functions in this commensal interaction.

Many functions listed in Table 5 may reflect the fact that these proteins may have multiple functions other than phosphorylation-induced signalling control in the host cell. Some of these proteins may mimic host protein functions. For example, it was suggested that CagA functions as a prokaryotic mimic of the eukaryotic Grb2-associated binder (Gab) adaptor protein [25] . Some of the predicted effectors may mimic singling proteins, such as HPK (histidine protein kinase) listed in Table 5, which is an important part of two-component signal transduction system that recognizes and transmits environmental signals [26] . Some known effectors induce protein expressions with increased expression of RNA polymerase [27] . It is not surprising to see a significant number of proteins in Table 5 are related to protein synthesis, such as RNA polymerase, elongation factor, and helicase. It is noted that CagA itself also contains an RNA polymerase domain based on a BLAST search. These connections also suggest the ancestor proteins of the predicted effectors. Many of the proteins listed in Table 5 are ancient house-keeping genes. The predicted effectors might have evolved from these house-keeping genes by mimicking the host genes. Furthermore, over evolution some of the effectors or their ancestors might have evolved into genes with different functions unrelated to host-pathogen interactions, such as EPIYA-motif containing proteins in archaea and metazoan.

### 4. HMMs based on EPIYA and SH2-binding motifs

**Figure 3:**
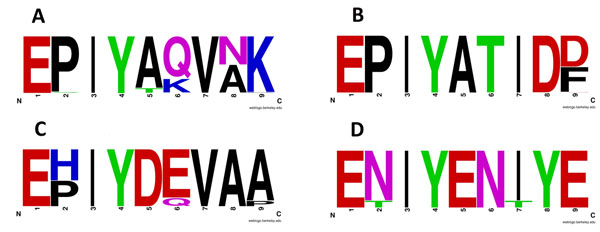
**Sequence logos for KK, R4, Tir and Tarp motifs** A: the logo was built with 1705 KK motif sequences extracted from 842 CagA protein sequences; B: the logo was built with 979 R4 motif sequences extracted from 842 CagA protein sequences; C: the logo was built with 20 Tir motif sequences extracted from 20 Tir protein sequences of *Escherichia Eoli* and *Citrobacter rodentium;* D: the logo was built with 16 Tarp motif sequences extracted from 7 Tarp protein sequences of *Chlamydie trachomatis*.

(1) Building HMM based on KK, R4, Tarp and Tir motifs It is known that biological effects of CagA induced by phosphorylation depend on the binding to the SH2 domain. Different combinations of the five amino acids after phosphorylated tyrosine (pY) will bind different SH2 domains and cause different downstream effects. There are two known motifs in CagA that could bind to SH2 domain - EPIYAKVNK and EPIYATIDD(F), which are referred to as KK motif and R4 motif [28,29] , respectively. For the Tir protein, we retrieved all sequences in *Escherichia coli* and *Citrobacter rodentium* with the pattern of EHIYDEVAA(P) and built a motif (named as Tir motif). We did the same for Tarp protein, yielding the motif ENIYENIYE (named as Tarp motif). We extracted sequences of known KK, R4, Tir and Tarp motifs in proteins CagA, Tir and Tarp (including their variants), and then built HMMs one by one. Figure 3 shows the sequence logo of each motif. Comparing with the sequence logo in Figure 1, the 9-mer motifs are more conserved and specific. This will help reduce the false positive rate in identifying putative effectors, while the downstream SH2 binding partners are also predicted at the same time.

(2) Search results by using HMMs based on KK, R4, Tarp, and Tir motifs Using HMMs based on KK, R4, Tarp and Tir motifs to search the protein sequences containing the EPIYA motif as described above, we found that the results are widespread in many species. In this paper we only focus on the results in bacteria and protista. As shown in Table 6, CagA KK (EPIYAKVNK) motif exists in some known phosphorylation effectors, e.g., Beps (BepD, BepE, BepH) and Tir. CagA R4 (EPIYATIDD) motif exists in Tarp. Both KK and R4 motifs exist in ankyrin. Among 8 proteins for building our EPIYA-motif based HMM, BepF is the only one containing neither KK nor R4 motif. The Tir protein just have one motif -Tir motif in Table 1, while BAF52548 (Tir of *E. coli)* contains two motifs, i.e., Tir (EHIYDEVAA) motif and EPIYAKIQR, similar to the KK motif. Tarp protein (YP_001654788) of *Chlamydia trachomatis* contains not only the Tarp motif (ENIYENIYE), but also another motif ENIYESIDD, which is similar to the R4 motif. It reveals that although these proteins are not similar in global sequences (weak similarity exists between Beps sequences), they share the same or similar motifs with significant functional relationships.

**Table 6:** Sequences containing KK and R4 motifs in known effectors

KK Motif	Species	Protein	pY prosition	Locus
**EPIYAKVNK**	*H.pylori*	cagA	Y-899	NP_207343

**EPIYTQVAK**	*H.pylori*	cagA	Y-918	NP_207343

**EPIYAKIQR**	*E.coli*	Tir	Y-477	BAF52548

**EPIYATVKK**	*Anaplasma phagocytophilum*	Ankyrin	Y-1094	ABB84853

**EPLYAQVNK**	*Bartonella henselae*	BepD protein	Y-28	YP_034066

**EPLYATVNK**	*Bartonella henselae*	BepE protein	Y-33	YP_034067

**EDLYATVGA**	*Anaplasma phagocytophilum*	Ankyrin	Y-1024	ABB84853

R4 **Motif**	**Species**	**Protein**	**pY prosition**	**Locus**

**EPIYATIDD**	*H.pylori*	cagA	Y-972	NP_207343

**ENIYESIDD**	*Chlamydia trachomatis*	Tarp	Y-189	YP_001654788

**ESIYEEIKD**	*Anaplasma phagocytophilum*	Ankyrin	Y-990	ABB84853

### 5. Prediction of new effectors

Based on the above HMMs of KK, R4, Tir and Tarp motifs, we predicted some new pathogen effectors (Additional File 3) and we assessed them based on the literature. The details of predicted effectors have been listed in Additional File 4, 5, 6, 7.

(1) *Bartonella tribocorum:* Since BepH contains the EPLYAQVNK (YP_001610013, Y-8) motif (KK motif), we predicted it as a phosphorylation effector like BepD-F secretory proteins.

(2) *Lawsonia intracellularis: Lawsonia intracellularis* is an obligate intracellular bacterial pathogen, which infects a wide range of animals, mainly pigs, and causes proliferative enteropathy - a type of contagious diseases [30,31] . Its symptoms are acute, including diarrhea, loss of appetite and stunting. After an initial close association with the cell membrane of the enterocytes, *Lawsonia intracellularis* is endocytosed into host cell [32] . Infected host cells are inhibited in maturation, continue to undergo mitosis and proliferation, and at last form hyperplastic crypts, but the mechanism is unknown [33] . The genome sequence of *Lawsonia intracellularis* indicates that it may possess a type III secretion system, which may assist the bacterium during cell invasion and evasion of the host's immune system and could be a mechanism for inducing cellular proliferation [34,35] , but its effectors secreted by T3SS was never reported. Current database contains 20 proteins of *Lawsonia intracellularis* with the EPIYA motif and all of them are from strain PHE/MN1-00. The maximum sequence identity between any two of these 20 proteins is 22% and most of them are enzymes, e.g. ATP synthase. Among them, in the HMM search result by using the R4 motif, we found that hypothetical protein L10666 (YP_595041) contains two copies of EPIYA motif (EPIYAEIKT Y-149, EPIYAEIKT Y-186), which are similar to the R4 and Tir motifs, respectively. Thus, we speculate that this protein might be the effector of *Lawsonia intracellularis* to interact with intestinal epithelial cells.

(3) *Ehrlichia sp.:* It belongs to the same family *Ehrilichiaceae* as *Anaplasma*[36] . Ankyrin of *Ehrlichia sp.* and ankyrin of *Anaplasma* share 89% sequence identity. Ankyrin (T08612) of *Ehrlichia sp.* contains six copies of 9-mer motifs including the KK and R4 motifs, and thus it is a likely effector of *Ehlichia* to interact with host.

(4) *Wolbachia: Wolbachia* belongs to *Rickettsiales. Wolbachia* is a symbiotic bacterium existing in the sex organ of many insects. Though ankyrin (AAY54257) of *Wolbachia* and ankryin of *Anaplasma* share only 15% sequence identity, they contain almost exactly the same motifs. Hypothetical protein WD0942 (NP_966676), which is not similar to ankyrin in sequence, has two motifs, and one of them is EPIYATVPK(Y-318) similar the KK motif. EsorChan1 (AAP34173) contains the motif EPIYDEVYD (Y-77) similar to the Tir motif. Therefore, the above three proteins, especially the first two, are potential effectors of *Wolbachia*[37] .

(5) *Pasterurella multocida:* As the major pathogen to cause swine infectious atrophic rhinitis, it secretes toxin filamentous hemagglutinin containing six copies of EPIYA motif. Based on the BLAST search results, we found that the filamentous hemagglutinin (AAK61595) of *Pasterurella multocida,* filamentous hemagglutinin of *Bordetella pertussis* and *Bordetella Parapertussis* share ~30% sequence identity [3840]. Filamentous hemagglutinin, the major virulence factor of *Bordetella pertussis,* not only has adhesion function, but also plays a critical role in immunomodulation. Since filamentous hemagglutinin has the sequences EDIYATINK (Y-2792), which is similar to the KK motif, EHIYADIRD (Y-2550) and ENLYAEISD (Y-2651), both of which are similar to the R4 motif, and EHLYAEINE (Y-2387), which is similar to the Tir motif, we suggest that filamentous hemagglutinin being the effector of *Pasterurella multocida* and it might be secreted by the TPS (Two-Partner Secretion) system [39] . PfhB2 (NP_244996) has four sequences that are similar to KK, R4 and Tir motifs, and thus it might be another candidate of effector in *Pasterurella multocida.*

(6) *Haemophilus ducreyi: Haemophilus ducreyi* is a facultative anaerobic Gram- negative coccobacillus and could cause the sexually transmitted disease chancroid. Large supernatant protein2 (NP_873623) of *Haemophilus ducreyi* has six copies of EPIYA motif. Its sequence and filamentous hemagglutinin of *Bordetella pertussis* share 41% sequence identity. Its sequences EPVYADLHF and EPVYADLRF are similar to the R4 motif. Hence, we suggest large supernatant protein2 (NP_873623) is a potential effector of *Haemophilus ducreyi* and it could be secreted by T4SS [41] . The effector can lead to immunosuppression, inhibition of proliferation, and permanent changes in host cells [42-44] .

(7) *Haemophilus somnus: Haemophilus somnus* can survive in host cells and is the cause of a variety of systemic diseases in cattle, including thrombotic-meningoencephalitis, pneumonia, arthritis, myocarditis, septicemia and other reproductive diseases [45,46] . Cysteine protease domain YopT-type (YP_001784809) and filamentous hemagglutinin of *Bordetella pertussis* share 42% sequence identity. The sequence EPIYATLDK (Y-2933) in YP_001784809 is similar to the KK motif, EHIYEQIGE (Y-2358) similar to the Tarp motif, and EPVYDKVSA (Y-2287) similar to the Tir motif. Thus, YP_001784809 might be the effector of *Haemophilus somnus* to cause immunosuppression [47] .

(8) *Chlamydophila pneumonia:* Hypothetical protein CPj0472 (NP_300527) contains three copies of EPIYA motif. EPIYANTPE (Y-647) is similar to the KK motif, EPIYEEIGG (Y-346) is similar to the Tir motif and EPIYDEIPW (Y-681) is similar to the R4 motif. Although we did not find any similar protein through BLAST search, hypothetical protein CPj0472 (NP_300527) is a good candidate for the effector of *Chlamydophila pneumonia.*

(9) *Leishmania major: Leishmania major* could parasitize into phagocyte of human or other mammals and is responsible for the disease leishmaniasis, which is a serious zoonosis. *Leishmania major* have 6 proteins containing at least two copies of EPIYA motif and 4 of them are proteins with unknown functions. Among these 6 proteins, Cytochrome C oxidase subunit VI (XP_001683136) contains two copies of EPIYA motif. One is at position Y-107 with sequence EPLYQPVKK, which is similar to the KK motif. Another one is at position Y-130 with sequence EPLYDVDAA, which is similar to the Tir motif. Hence, XP_001683136 might be an effector. Hypothetical protein (XP_001686159) has three copies of EPIYA motif and the sequences are all EPLYAVTIE, which is similar to KK and R4 motifs. Hypothetical protein (XP_001686160) also has three copies of EPIYA motif and the sequences are all EPLYAVTID, which is similar to the R4 motif. In addition, hypothetical protein XP_001686159 and XP_001686160 share 43% sequence identity. Hypothetical protein (XP_001686356) has 29 copies EPIYA motif (the one with most EPIYA motifs in our data) and all sequences are the same as EPLYAVTLE, which is similar to the R4 motif. Microtubule-associated protein (XP_001687515) contains two copies of EPIYA motif. One is at Y-1543 and another is at Y-1589. The sequences for both of them are ESIYAKDYK, which is similar to the KK motif. Thus, we predict hypothetical protein (XP_001686159), hypothetical protein (XP_001686160), hypothetical protein (XP_001686356) and Microtubule-associated protein (XP_001687515) might also be the effectors of *Leishmania major.* For another potential effector hypothetical protein (XP_001683914), although it contains two copies of EPIYA motif (ESLYE is at Y-1006 and EHLYD is at Y-1047), they are not similar to KK, R4, Tarp or Tir motif and hence less likely to be an effector than the above five proteins.

(10) *Plasmodium falciparum: Plasmodium falciparum* can invade human liver cells and RBC to cause dangerous infection malaria. It contains many proteins with the EPIYA motif and 47 proteins with at least two copies of EPYIA motif. Among them, Plasmodium exported protein (XP_001347309) has three copies of motif which are all similar to the Tarp motif. The sequences and the corresponding pY sites are ESIYKNKLK (Y-331), ESIYKNKLK (Y-359), and ESIYKNKLE (Y-387). Thus we predict it as the effector of *Plasmodium falciparum.* Conserved *Plasmodium* protein (XP_001347469) has eight copies of EPIYA motif, RNA pseudouridylate synthase (XP_001350676) has nine copies of EPIYA motifs and hypothetical protein (XP_001351018) has three copies of EPIYA motif, but none of them contains any of KK, R4, Tir and Tarp motifs, and therefore is less likely to be the effector than Plasmodium exported protein (XP_001347309).

### 6. Protein subcellular localization prediction

We applied the subcellular localization prediction for all the predicted bacterial effectors above by using CELLO v.2.5 [48] (http://cello.life.nctu.edu.tw). All the associated bacteria are “gram negative”. As a real effector should be secreted from a gram-negative bacterium and then enter a eukaryote host, we perform subcellular localization by using both gram-negative bacterium and eukaryote, respectively. When using gram-negative bacteria as hosting species, 9 out 11 effectors were predicted as extracellular or outer-membrane (Additional File 8). When reapplying the prediction by choosing eukaryotes as the organisms, all 11 effectors are predicted to have nuclear localization (Additional File 8). The above results show that most of our predicted effectors have expected localization attributes as effectors, which provides some supporting evidence for our effector predictions.

## Conclusions

In this paper, we showed that the EPIYA motif might be a ubiquitous functional site for effectors that play an important role in pathogenicity for mediating host-pathogen interactions. Most known effectors have more than one copy of EPIYA motif. The predicted effector sequences of pathogens from the same genus are likely homologous, and those from different genuses are rarely homologous although they often share common motifs. Most pathogens are intracellular bacteria or long-term chronic infection of extracellular bacteria, e.g., *H. pylori.* Usually effectors are secreted by T3SS or T4SS to enter host cells, and then interfere signal transduction pathway of the host cell to disturb host cell functions, which mainly involve actin polymerization, cell proliferation, apoptosis and immunosuppression, so as to improve the abilities of survival and propagation of microorganism with host-pathogen interaction.

Our study predicted many putative effectors. We grouped the phosphorylated EPIYA motifs into four types, KK, R4, Tir and Tarp based on the sequence features of the five amino acids after Y, and then we used them individually to build the HMM. After using the HMMs to search our database and considering the known pathogenic characteristics of pathogens, we predicted some effectors of bacteria and also suggested that using our method will discover more effectors with the EPIYA motif. Besides the discovery in bacteria, we also found that there were many protein sequences containing the EPIYA motif in protist pathogens. Intracellular protozoan parasites can live in host cells to survive and reproduce by subverting of host cell signalling [19] , to induce downstream effects, e.g., inhibiting apoptosis of host cells, restructuring of the host cell cytoskeleton, and so on. However, the pathogen mediators responsible for this modulation are still unknown [20] . Based on this study, we hypothesize that during the interaction process between protist and host, there is a secretion system that can secrete effectors to disturb the signal transduction pathway of infected host and to control the apoptosis of host cells.

Our predictions provide useful hypotheses for further studies on exploring pathogenic mechanisms in the host-pathogen interactions. It also has the scientific and clinical implications for prevention and treatment of infectious diseases, as it may provide some guidance for vaccine/drug development. Having said that, it is noted that the EPIYA-motif containing protein does not exist in all intracellular bacteria, and therefore EPIYA-motif mediating interaction is only one type of various host- pathogen mechanisms. Furthermore, our prediction result is based on computation and definitely contains false positives, and thus it requires further experimental validations.

## Methods

**Figure 4:**
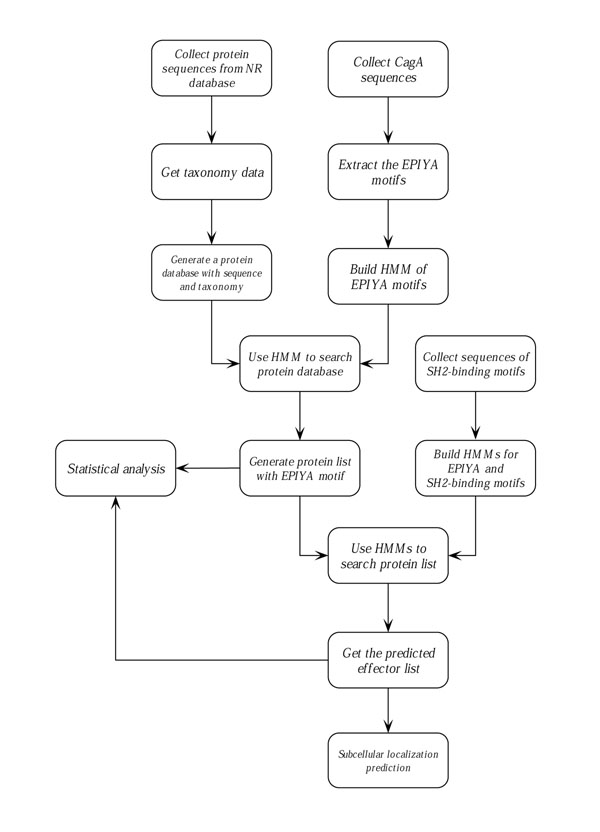
Workflow of the whole project.

Figure 4 shows the overall workflow of this project. The major modules and steps are described as follows:

### Data Sources

1. Protein sequence data: We used the NR (non-redundant) protein database at the National Center for Biotechnology Information (NCBI) in this study. All protein sequences in the FASTA format were downloaded from the NCBI site (ftp://ftp.ncbi.nih.gov/blast/db/FASTA/nr.gz; as of July 6^th^ 2009; 9,216,047 sequences). We excluded “other” sequences and “unclassified” sequences” in the database (as labelled in http://www.ncbi.nlm.nih.gov/Taxonomy/Browser/wwwtax.cgi?mode=Root).

2. Taxonomy data: The taxonomy data was obtained from the NCBI website (http://www.ncbi.nlm.nih.gov/Taxonomy/txstat.cgi; as of July 6^th^ 2009).

### Hidden Markov model

A hidden Markov model was built by using Hmmer 2.3.2 [49] (http://hmmer.janelia.org). We used selected sequences to run the command hmmbuild.exe for building and calibrating the HMM. We then used the HMM to run the command hmmsearch.exe for searching protein sequences. We used a natural cutoff of HMM score such that the last of the all known motifs is retrieved.

### Data analysis

We used Perl (release ActivePerl 5.8.8) as the programming language to analyse the data and build the database. We applied SAS 9.0 (http://www.sas.com) as the statistical analysis tool and chose p<0.01 as the significant threshold.

### Sequences comparison

BioEdit 7.0 (http://www.mbio.ncsu.edu/BioEdit/bioedit.html), Lasergene 7 (http://www.dnastar.com/products/lasergene.php), and Blast [50] (http://blast.ncbi.nlm.nih.gov/Blast.cgi) were used to compare and analyse the protein sequences. Sequence logos were constructed using Weblogo [51] .

## Authors' contributions

SX conceived the study, carried out the programming, built EPIYA-motif HMM, performed data analysis and drafted the manuscript. CZ built EPIYA-motif HMM, performed data analysis and drafted the manuscript. YM conceived the study. JG participated in data analysis. DX conceived the study, analyzed the results, and supervised the project. All authors revised and approved the final manuscript.

## Competing interests

The authors declare that they have no competing interests.

## Supplementary Material

Additional File 1 Top 10 genuses and species containing most proteins with at least two copies of EPIYA motif for each group. Click here for file

Additional File 2 List of proteins that contain at least 4 EPIYA motifs. Parenthesis under Lotus: number of sequences; “*”: effector confirmed by experiment; “Repeats”: occurrence of EPIYA motif in a protein sequence. Click here for file

Additional File 3 EPIYA motif, together with the corresponding functions of those effectors. *: predicted effectors; **: predicted motifs. Click here for file

Additional File 4 This file contains a list of sequences that are similar to the KK motif in bacteria and protista. Click here for file

Additional File 5 This file contains a list of sequences that are similar to the R4 motif in bacteria and protista. Click here for file

Additional File 6 This file contains a list of sequences that are similar to the Tarp motif in bacteria and protista. Click here for file

Additional File 7 This file contains a list of sequences that are similar to the Tir motif in bacteria and protista. Click here for file

Additional File 8 This file contains top three subcellular localization prediction results of each predicted effector using gram-negative bacterium or eukaryote as the hosting organism, respectively. The values indicate the confidence of the predictions and “*” represents most likely localization. Click here for file
