# Supplementary material for: Effector prediction in host-pathogen interaction based on a Markov model of a ubiquitous EPIYA motif
Source: BMC Genomics. 2010 Dec 1;11(Suppl 3):S1. doi: 10.1186/1471-2164-11-S3-S1 (PMC2999339; doi:10.1186/1471-2164-11-S3-S1)
Supplement: Additional File 1 — Top 10 genuses and species containing most proteins with at least two copies of EPIYA motif for each group. [file 1471-2164-11-S3-S1-S1.doc]

Additional File 1: Top 10 genuses and species containing most proteins with at least two copies of EPIYA motif for each group

| Group | rank by genus | occurrence | rank by species | occurrence |
| --- | --- | --- | --- | --- |
| **Archaea** | Methanococcus | 11 | Methanococcus maripaludis | 9 |
|  | Methanocaldococcus | 6 | Aciduliprofundum boonei | 4 |
|  | Aciduliprofundum | 4 | Halogeometricum borinquense | 4 |
|  | Halogeometricum | 4 | Methanocaldococcus jannaschii | 4 |
|  | Methanobrevibacter | 3 | Methanobrevibacter smithii | 3 |
|  | Methanosarcina | 3 | Halorubrum lacusprofundi | 2 |
|  | Thermococcus | 3 | Hyperthermus butylicus | 2 |
|  | Haloferax | 2 | Methanosarcina barkeri | 2 |
|  | Halorubrum | 2 | Methanospirillum hungatei | 2 |
|  | Hyperthermus | 2 | Archaeoglobus fulgidus | 1 |
| **Viruses** | Potyvirus | 14 | Bovine Viral Diarrhea Virus | 11 |
|  | Pestivirus | 11 | Zucchini yellow | 8 |
|  | Orthopoxvirus | 5 | Bean common | 5 |
|  | Simplexvirus | 5 | Grapevine virus | 5 |
|  | Vitivirus | 5 | Cowpox virus | 3 |
|  | Capripoxvirus | 4 | Lumpy skin | 2 |
|  | Yatapoxvirus | 3 | Papiine herpesvirus | 2 |
|  | Alphabaculovirus | 2 | Tanapox virus | 2 |
|  | T4-likeviruses | 2 | Acidianus filamentous | 1 |
|  | Alphapapillomavirus | 1 | Aeromonas phage | 1 |
| **Bacteria** | Helicobacter | 1024 | Helicobacter pylori | 1021 |
|  | Bacillus | 133 | Bacillus cereus | 78 |
|  | Clostridium | 102 | Anaplasma phagocytophilum | 46 |
|  | Anaplasma | 48 | Bacillus thuringiensis | 28 |
|  | Bacteroides | 23 | Clostridium botulinum | 28 |
|  | Vibrio | 17 | Clostridium perfringens | 20 |
|  | Lactobacillus | 16 | Cyanothece sp. | 11 |
|  | Cyanothece | 11 | Bacteroides sp. | 10 |
|  | Ureaplasma | 11 | Lactococcus lactis | 10 |
|  | Campylobacter | 10 | Chlamydia trachomatis | 9 |
| **Protista** | Plasmodium | 103 | Plasmodium falciparum | 47 |
|  | Tetrahymena | 35 | Tetrahymena thermophila | 35 |
|  | Paramecium | 26 | Paramecium tetraurelia | 26 |
|  | Entamoeba | 14 | Plasmodium yoelii | 19 |
|  | Leishmania | 14 | Trichomonas vaginalis | 14 |
|  | Trichomonas | 14 | Plasmodium vivax | 12 |
|  | Cryptosporidium | 11 | Plasmodium knowlesi | 11 |
|  | Giardia | 9 | Entamoeba dispar | 8 |
|  | Monosiga | 8 | Giardia lamblia | 8 |
|  | Theileria | 7 | Monosiga brevicollis | 8 |
| **Fungi** | Candida | 21 | Candida tropicalis | 9 |
|  | Aspergillus | 15 | Paracoccidioides brasiliensis | 9 |
|  | Pichia | 12 | Candida albicans | 8 |
|  | Paracoccidioides | 9 | Pichia stipitis | 6 |
|  | Ajellomyces | 7 | Vanderwaltozyma polyspora | 6 |
|  | Vanderwaltozyma | 6 | Ajellomyces capsulatus | 5 |
|  | Coccidioides | 5 | Cryptococcus neoformans | 5 |
|  | Filobasidiella | 5 | Saccharomyces cerevisiae | 5 |
|  | Saccharomyces | 5 | Aspergillus terreus | 4 |
|  | Debaryomyces | 4 | Candida dubliniensis | 4 |
| **Metazoa** | Drosophila | 178 | Homo sapiens | 69 |
|  | Homo | 69 | Mus musculus | 58 |
|  | Mus | 58 | Drosophila melanogaster | 41 |
|  | Pan | 30 | Pan troglodytes | 29 |
|  | Branchiostoma | 27 | Branchiostoma floridae | 27 |
|  | Caenorhabditis | 21 | Rattus norvegicus | 21 |
|  | Rattus | 21 | Canis lupus | 20 |
|  | Canis | 20 | Danio rerio | 17 |
|  | Danio | 17 | Drosophila pseudoobscura | 17 |
|  | Macaca | 16 | Drosophila persimilis | 15 |
| **Viridiplantae** | Oryza | 24 | Oryza sativa | 23 |
|  | Physcomitrella | 12 | Physcomitrella patens | 12 |
|  | Arabidopsis | 10 | Arabidopsis thaliana | 10 |
|  | Populus | 7 | Populus trichocarpa | 7 |
|  | Sorghum | 7 | Sorghum bicolor | 7 |
|  | Ricinus | 6 | Ricinus communis | 6 |
|  | Vitis | 6 | Vitis vinifera | 6 |
|  | Micromonas | 3 | Micromonas pusilla | 3 |
|  | Huperzia | 2 | Huperzia lucidula | 2 |
|  | Ostreococcus | 2 | Zea mays | 2 |
