# Supplementary material for: Effector prediction in host-pathogen interaction based on a Markov model of a ubiquitous EPIYA motif
Source: BMC Genomics. 2010 Dec 1;11(Suppl 3):S1. doi: 10.1186/1471-2164-11-S3-S1 (PMC2999339; doi:10.1186/1471-2164-11-S3-S1)
Supplement: Additional File 2 — List of proteins that contain at least 4 EPIYA motifs. Parenthesis under Lotus: number of sequences; “*”: effector confirmed by experiment; “Repeats”: occurrence of EPIYA motif in a protein sequence. [file 1471-2164-11-S3-S1-S2.doc]

Additional File 2: List of proteins with number of EPIYA motif repeats≥4

| **Name of protein** | **Group** | **Species** | **Repeats** | **Locus** |
| --- | --- | --- | --- | --- |
| CagA | Bacteria | *Helicobacter pylori* | 4-7 | (184 copies)* |
| Ankyrin | Bacteria | *Anaplasma phagocytophilum* | 4-8 | (42 copies)* |
| ankyrin repeat domain protein, putative | Bacteria | *Wolbachia endosymbiont* | 4 | ZP_03334563 |
| ankyrin-like protein, 160K | Bacteria | *Ehrlichia sp.* | 6 | T08612 |
| BepD protein | Bacteria | *Bartonella grahamii* | 4 | YP_002972372# |
| cysteine protease domain-containing protein | Bacteria | *Haemophilus somnus* | 5 | YP_001784809 |
| Chain A | Bacteria | *Escherichia coli* | 5 | 3CMT_A |
| Chain A | Bacteria | *Escherichia coli* | 6 | 3CMU_A |
| Chain A | Bacteria | *Escherichia coli* | 4 | 3CMV_A |
| hypothetical protein L8106_16549 | Bacteria | *Lyngbya sp.* | 5 | ZP_01620341 |
| hypothetical protein L8106_30180 | Bacteria | *Lyngbya sp.* | 5 | ZP_01622571 |
| hypothetical protein | Bacteria | *Dorea longicatena* | 7 | ZP_01994940 |
| hypothetical protein Btr_1705 | Bacteria | *Bartonella tribocorum* | 5 | YP_001610012 |
| hypothetical protein AMF_343 | Bacteria | *Anaplasma marginale* | 4 | YP_002563468 |
| hypothetical protein | Bacteria | *Actinomyces coleocanis* | 4 | ZP_03924750 |
| hypothetical protein gll2226 | Bacteria | *Gloeobacter violaceus* | 5 | NP_925172 |
| hypothetical protein BH13410 | Bacteria | *Bartonella henselae* | 12 | YP_034066 |
| hypothetical protein BH13430 | Bacteria | *Bartonella henselae* | 4 | YP_034068 |
| hypothetical protein AM470 | Bacteria | *Anaplasma marginale* | 4 | YP_153762 |
| kinesin light chain-like protein | Bacteria | *Lyngbya sp.* | 8 | ZP_01624728 |
| large supernatant protein | Bacteria | *Haemophilus ducreyi* | 6 | NP_873623 |
| NB-ARC domain protein | Bacteria | *Cyanothece sp.* | 8 | ZP_03153368 |
| PfhB2 | Bacteria | *Pasteurella multocida* | 4 | NP_244996 |
| putative filamentous hemagglutinin | Bacteria | *Pasteurella multocida* | 6 | AAK61595 |
| Tarp | Bacteria | *Chlamydia trachomatis* | 6 | YP_001654788* |
| Tarp | Bacteria | *Chlamydia trachomatis* | 6 | YP_001653800* |
| tetratricopeptide TPR_2 | Bacteria | *Trichodesmium erythraeum* | 14 | YP_720940 |
| tetratricopeptide repeat family | Bacteria | *Stigmatella aurantiaca* | 7 | ZP_01462084 |
| tetratricopeptide repeat family | Bacteria | *Stigmatella aurantiaca* | 13 | ZP_01466552 |
| TPR repeat-containing protein | Bacteria | *Acaryochloris marina* | 4 | YP_001522281 |
| TPR repeat-containing protein | Bacteria | *Cyanothece sp.* | 7 | ZP_03144860 |
| TPR repeat-containing protein | Bacteria | *Cyanothece sp.* | 4 | ZP_03153325 |
| TPR repeat-containing protein | Bacteria | *Cyanothece sp.* | 5 | ZP_03156190 |
| Tetratricopeptide TPR_2 repeat protein | Bacteria | *Cyanothece sp.* | 4 | YP_002371949 |
| Tetratricopeptide repeat family | Bacteria | *Microcoleus chthonoplastes* | 5 | YP_002619484 |
| tetratricopeptide repeat protein | Bacteria | *Haliangium ochraceum* | 4 | ZP_03879805 |
| tetratricopeptide repeat protein | Bacteria | *Haliangium ochraceum* | 5 | ZP_03880192 |
| TPR repeat-containing protein | Bacteria | *Nitrobacter winogradskyi* | 4 | YP_316787 |
| tetratricopeptide TPR_4 | Bacteria | *Nitrosospira multiformis* | 4 | YP_412276 |
| tetratricopeptide TPR_2 | Bacteria | *Nitrobacter hamburgensis* | 5 | YP_578440 |
| conserved Plasmodium protein | Protista | *Plasmodium falciparum* | 8 | XP_001347469 |
| hypothetical protein TTHERM_01044690 | Protista | *Tetrahymena thermophila* | 4 | XP_001030642 |
| hypothetical protein | Protista | *Plasmodium falciparum* | 5 | XP_001351018 |
| hypothetical protein | Protista | *Leishmania infantum* | 10 | XP_001468598 |
| hypothetical protein | Protista | *Leishmania major* | 29 | XP_001686356 |
| RNA pseudouridylate synthase, putative | Protista | *Plasmodium falciparum* | 9 | XP_001350676 |
| AGAP007452-PA | Metazoa | *Anopheles gambiae* | 5 | XP_001687921 |
| GA17694 | Metazoa | *Drosophila pseudoobscura* | 4 | XP_001352822 |
| GK17141 | Metazoa | *Drosophila willistoni* | 4 | XP_002061710 |
| GL20865 | Metazoa | *Drosophila persimilis* | 4 | XP_002025727 |
| hypothetical protein | Metazoa | *Nematostella vectensis* | 4 | XP_001623017 |
| hypothetical protein | Metazoa | *Nematostella vectensis* | 5 | XP_001632861 |
| hypothetical protein | Metazoa | *Nematostella vectensis* | 14 | XP_001636029 |
| hypothetical protein BRAFLDRAFT_97610 | Metazoa | *Branchiostoma floridae* | 13 | XP_002235088 |
| hypothetical protein | Metazoa | *Hydra magnipapillata* | 4 | XP_002160378 |
| kinase different kind | Metazoa | *Monodelphis domestica* | 7 | XP_001381219 |
| PREDICTED: similar to Cuticular protein 76Bd CG9299-PB | Metazoa | *Acyrthosiphon pisum* | 4 | XP_001944766 |
| PREDICTED: similar to MGC84161 protein | Metazoa | *Strongylocentrotus purpuratus* | 4 | XP_783664 |
| hypothetical protein OsJ_05440 | Viridiplantae | *Oryza sativa* | 4 | EEE56337 |
| 135R protein | Viruses | *Yaba-like disease virus* | 4 | NP_073520 |
| VARV B22R | Viruses | *Tanapox virus* | 4 | ABQ43766 |
| VARV B22R | Viruses | *Tanapox virus* | 4 | YP_001497131 |
