# Supplementary material for: Effector prediction in host-pathogen interaction based on a Markov model of a ubiquitous EPIYA motif
Source: BMC Genomics. 2010 Dec 1;11(Suppl 3):S1. doi: 10.1186/1471-2164-11-S3-S1 (PMC2999339; doi:10.1186/1471-2164-11-S3-S1)
Supplement: Additional File 3 — EPIYA motif, together with the corresponding functions of those effectors. *: predicted effectors; **: predicted motifs. [file 1471-2164-11-S3-S1-S3.doc]

Additional File 3: EPIYA-motif containing effectors and corresponding functions of pathogenesis

| **Protein**  **name** | **Species** | **Secretion system** | **pY sites** | **Pathogenic process** | **Associated diseases** | **motif and pY position** | **Locus of Protein**  **and Reference** |
| --- | --- | --- | --- | --- | --- | --- | --- |
| CagA | *H.pylori* | T4SS | 3 | rearrangement of cytoskeleton, NF-kB activation and inhibiting apoptosis | gastritis, peptic ulcer, gastric cancer | EPIYAKVNK Y-899 EPIYTQVAK Y-918  EPIYATIDD Y-972 | NP_207343  [7, 8, 10, 54-58] |
| Tir | E.coli | T3SS | 2 | actin polymerization | diarrhea | EHIYDQVAA Y-471 EPIYAKIQR** Y-481 | BAF52548  [51, 59, 60] |
| Tir | C.rodentium | T3SS | 1 | actin polymerization | diarrhea | EPIYDEVAP Y-468 | AAL06376  [52] |
| Tarp | C.trachomatis | T3SS | 6 | actin polymerization | trachoma and sexually transmitted disease | ENIYENIYE Y-136 ENIYESIDD** Y-189  ENIYENIYE Y-238 ENIYESIDD** Y-291  ENIYESIDD** Y-341 ENIYENIYE Y-390 | YP_001654788  [53, 61, 62] |
| Ankyrin | A.phagocytophilum | T4SS | 7 | inhibiting respiratory burst and apoptosis | human granulocytic anaplasmosis | ESIYEEIKD Y-940 ESIYEEIKD Y-967  ESIYEEIKD Y-994 EDLYATVGA Y-1028  ESIYADPFD Y-1056 ESIYADPFA Y-1074  EPIYATVKK Y-1098 | ABB84853  [12, 13, 36] |
| BepD | B.henselae | T4SS | 12 | rearrangement of cytoskeleton, NF-kB activation and inhibiting apoptosis | cat scratch disease and bacillary peliosis | EPLYAQVNK Y-32 ETIYAPQNP Y-52  ETIYAPQKP Y-62 NPLYEGVGG Y-114  EHLYAELEF Y-134 NPLYEGVGS Y-176  EPLYAQVNK Y-211 ETIYAPQNP Y-231  ETIYAPQKP Y-241 NPLYEGVGG Y-293  EHLYAELEF Y-313 NPLYEGVGP Y-355 | YP_034066  [14-16, 63] |
| BepE | B.henselae | T4SS | 3 | rearrangement of cytoskeleton, NF-kB activation and inhibiting apoptosis | cat scratch disease and bacillary peliosis | EPLYATVNK Y-37 ETIYTTVSS Y-91  ETLYAEVAM Y-129 | YP_034067 |
| BepF | B.henselae | T4SS | 4 | rearrangement of cytoskeleton, NF-kB activation and inhibiting apoptosis | cat scratch disease and bacillary peliosis | EPLYATPLP Y-213 EPLYATPLP Y-241  EPLYATPLP Y-269 EPLYATAAP Y-297 | YP_034068 |
| BepH* | A.tribocorum | T4SS | 1 | rearrangement of cytoskeleton, NF-kB activation and inhibiting apoptosis | cat scratch disease and bacillary peliosis | EPLYAQVNK Y-9 | YP_001610013 |
| Ankyrin-like protein* | Ehrlichia sp. | T4SS | 6 | inhibiting respiratory burst and apoptosis | human granulocytic anaplasmosis | ESIYEEIKD Y-456 ESIYEEIKD Y-483  EDLYATVGA Y-517 ESIYADPFD Y-545  ESIYADPFA Y-563 EPIYATVKK Y-587 | T08612  [36] |
| Ankyrin* | W. pipientis | T4SS | 3 | reproduction control | cytoplasmic incompatibility and parthenogenesis | EPIYAEVYD Y-193 ESIYAEIYD Y-219  EPIYARVDL Y-277 | AAY54257  [37, 64-67] |
| protein WD0942* | W.endosymbiont | T4SS | 2 | reproduction control | cytoplasmic incompatibility and parthenogenesis | NPLYEQRED Y-270 EPIYATVPK Y-318 | NP_966676  [64, 68] |
| EsorChan1* | W.endosymbiont | T4SS | 1 | reproduction control | cytoplasmic incompatibility and parthenogenesis | EPIYDEVYD Y-77 | AAP34173 |
| hypothetical protein LI0666 | L. intracellularis | T3SS | 2 | accelerating proliferation and inhibiting apoptosis | porcine proliferative enteropathies | EPIYAELDF Y-149 EPIYAEIKT Y-186 | YP_595041  [30, 33, 34, 69, 70] |
| hypothetical protein CPj0472* | C.pneumoniae | T3SS | 3 | actin polymerization | respiratory tract and pulmonary infections | EPIYEEIGG Y-346 EPIYANTPE Y-647  EPIYDEIPW Y-681 | NP_300527  [71, 72] |
| large supernatant protein 2* | H.ducreyi | T4SS | 6 | adhesion, regulating cell cycle, immunosuppression and inhibiting proliferation | chancroid | EPVYADLHF Y-3398 EPVYADLRF Y-3478  EPVYADLHF Y-3717 EPVYADLRF Y-3797  EPVYADLHF Y-4036 EPVYADLRF Y-4116 | NP_873623  [41-44] |
| cysteine protease domain, YopT-type* | H.somnus | T4SS | 5 | adhesion and immunosuppression | respiratory disease complex | EPVYDKVSA Y-2287 EHIYEQIGE Y-2358  EHIYEQIGE Y-2428 EHIYEQIGE Y-2498  EPIYATLDK Y-2933 | YP_001784809  [45-47] |
| filamentous hemagglutinin* | P.multocida | TPS? | 6 | adhesion and immunosuppression | atrophic rhinitis | NPIYESADA Y-2319 EHLYAEINE Y-2387  EHIYTDISD Y-2451 EHIYADIRD Y-2551  ENLYAEISD Y-2651 EDIYATINK Y-2792 | AAK61595  [39, 73-75] |
| PfhB2 | P.multocida | TPS? | 4 | adhesion and immunosuppression | atrophic rhinitis | NPIYESADA Y-2319 EHLYAEINE Y-2387  EHIYTDISD Y-2451 EPVYASVDK Y-3213 | NP_244996 |
| cytochrome C oxidase subunit VI | L.major | ? | 2 | immunosuppression and inhibiting apoptosis | leishmaniasis | EPLYQPVKK Y-107 EPLYDVDAA Y-130 | XP_001683136 |
| hypothetical protein | L.major | ？ | 29 | immunosuppression and inhibiting apoptosis | leishmaniasis | EPLYAVTLE  (29 in total, see Additional File 2) | XP_001686356 |
| hypothetical protein | L.major | ？ | 3 | immunosuppression and inhibiting apoptosis | leishmaniasis | EPLYAVTIE Y-473 EPLYAVTIE Y-695  EPLYAVTIE Y-917 | XP_001686159 |
| hypothetical protein | L.major | ？ | 3 | immunosuppression and inhibiting apoptosis | leishmaniasis | EPLYAVTID Y-668 EPLYAVTID Y-799  EPLYAVTLN Y-3002 | XP_001686160 |
| conserved Plasmodium protein | P.falciparum | ？ | 8 | immunosuppression and inhibiting apoptosis | falciparum malaria | EKIYDDNNK Y-201 EKIYDDNNK Y-210  EKIYDDNNK Y-219 EKIYDDNNK Y-228  EKIYDDNNK Y-237 EKIYDDNNN Y-246  KVIYKNIYS Y-1198 KQIYEKEYN Y-1477 | XP_001347469 |
| Plasmodium exported protein | P.falciparum | ？ | 3 | immunosuppression and inhibiting apoptosis | falciparum malaria | ESIYKNKLE Y-331 ESIYKNKLK Y-359  ESIYKNKLK Y-387 | XP_001347309 |
