# Supplementary material for: Effector prediction in host-pathogen interaction based on a Markov model of a ubiquitous EPIYA motif
Source: BMC Genomics. 2010 Dec 1;11(Suppl 3):S1. doi: 10.1186/1471-2164-11-S3-S1 (PMC2999339; doi:10.1186/1471-2164-11-S3-S1)
Supplement: Additional File 4 — This file contains a list of sequences that are similar to the KK motif in bacteria and protista. [file 1471-2164-11-S3-S1-S4.doc]

Additional File 4: KK-like motif sequences in bacteria and protista

| **KK motif** | **Species** | **Protein** | **pY position** | **Locus** |
| --- | --- | --- | --- | --- |
| **EPIYAKVNK** | *H.pylori* | cagA | Y-899 | NP_207343 |
| **EPIYAKIQR** | *Escherichia coli* | Tir | Y-481 | BAF52548 |
| **EPLYAQVNK** | *Bartonella tribocorum* | BepH protein | Y-8 | YP_001610013 |
| **EPIYATVPK** | *Wolbachiaendosymbiont* | hypothetical protein WD0942 | Y-318 | NP_966676 |
| **EPIYATIPK** | *Wolbachia endosymbiont* | hypothetical protein WPa_0152 | Y-194 | YP_001974970 |
| **EPIYATVKK** | *Ehrlichia sp.* | ankyrin-like protein, 160K | Y-587 | T08612 |
| **EHIYAQNSK** | *Helicobacter hepaticus* | hypothetical protein HH1030 | Y-408 | NP_860561 |
| **EPLYAELRK** | *Ochrobactrum anthropi* | hypothetical protein Oant_4474 | Y-264 | YP_001373002 |
| **ETIYAKLCK** | *Helicobacter acinonychis* | conserved hypothetical protein fragment | Y-30 | YP_664436 |
| **EHIYQQVSK** | *Escherichia coli* | transcription-repair coupling factor | Y-245 | NP_287248 |
| **EEIYAKVVD** | *Helicobacter acinonychis* | typeIIS restriction enzyme | Y-50 | YP_664039 |
| **EDIYATINK** | *Pasteurella multocida* | filamentous hemagglutinin | Y-2792 | AAK61595 |
| **EPIYATLDK** | *Haemophilus somnus* | cysteine protease domain, YopT-type | Y-2933 | YP_001784809 |
| **EPIYANTPE** | *Chlamydophila pneumoniae* | hypothetical protein CPj0472 | Y-647 | NP_300527 |
| **EPVYASVDK** | *Pasteurella multocida* | PfhB2 | Y-3213 | NP_244996 |
| **EALYAKVNK** | *Bartonella tribocorum* | hypothetical protein Btr_1705 | Y-22 | YP_001610012 |
| **ENIYAPQNP** | *Bartonella tribocorum* | hypothetical protein Btr_1705 | Y-69 | YP_001610012 |
| **EALYAKVNK** | *Bartonella tribocorum* | hypothetical protein Btr_1705 | Y-169 | YP_001610012 |
| **ETIYANIEK** | *Agrobacterium vitis* | acrB/acrD/acrF family protein | Y-411 | YP_002547375 |
| **EQIYANHSE** | *Tetrahymena thermophila* | HECT domain and Rcc1-like domain-containing protein | Y-1544 | XP_001017227 |
| **EPIYSTNKK** | *Tetrahymena thermophila* | hypothetical protein | Y-470 | XP_001019273 |
| **EDIYAKSTE** | *Trichomonas vaginalis* | hypothetical protein | Y-1489 | XP_001306505 |
| **EPIYTATKK** | *Trichomonas vaginalis* | hypothetical protein | Y-3434 | XP_001324654 |
| **EHIYNNIKK** | *Plasmodium falciparum* | hypothetical protein | Y-17 | XP_001351358 |
| **EHIYENVEE** | *Plasmodium falciparum* | hypothetical protein | Y-205 | XP_001351017 |
| **EPIYDEKQN** | *Plasmodium falciparum* | cysteine repeat modular protein 2 homologue | Y-689 | XP_001349085 |
| **EEIYQRNNK** | *Plasmodium falciparum* | hypothetical protein | Y-1099 | XP_001350310 |
| **EKIYTNNNK** | *Plasmodium falciparum* | conserved Plasmodium protein | Y-619 | XP_001347469 |
| **ESIYTQVLK** | *Paramecium tetraurelia* | hypothetical protein | Y-603 | XP_001438132 |
| **EPIYQQEEI** | *Paramecium tetraurelia* | hypothetical protein | Y-1751 | XP_001453558 |
| **NPIYINLEE** | *Paramecium tetraurelia* | hypothetical protein | Y-2601 | XP_001453558 |
| **EPLYAVTIE** | *Leishmania infantum* | hypothetical protein | Y-473 | XP_001468457 |
| **EPLYAVTIE** | *Leishmania infantum* | hypothetical protein | Y-695 | XP_001468457 |
| **EPLYAVTIE** | *Leishmania infantum* | hypothetical protein | Y-917 | XP_001468457 |
| **EPLYAVTIK** | *Leishmania braziliensis* | hypothetical protein | Y-143 | XP_001564708 |
| **EPLYQPVKK** | *Leishmania braziliensis* | cytochrome C oxidase subunit VI | Y-107 | XP_001564903 |
| **EDIYAITEK** | *Plasmodium vivax* | hypothetical protein | Y-5982 | XP_001613734 |
| **ESIYAKDYK** | *Leishmania major* | microtubule-associated protein | Y-1546 | XP_001687515 |
| **ESIYAKDYK** | *Leishmania major* | microtubule-associated protein | Y-1592 | XP_001687515 |
| **EPLYQPVKK** | *Leishmania major* | cytochrome C oxidase subunit VI | Y-107 | XP_001683136 |
| **EPLYAASEK** | *Leishmania major* | hypothetical protein | Y-167 | XP_001686307 |
| **EPLYASVAE** | *Leishmania major* | dynein heaVy chain | Y-3033 | XP_001686494 |
| **EPIYQKLRE** | *Entamoeba dispar* | hypothetical protein | Y-882 | XP_001741812 |
| **EPIYELIKE** | *Entamoeba histolytica* | pumilio family RNA-binding protein | Y-118 | XP_657035 |
| **ESIYKRFNK** | *Entamoeba histolytica* | hypothetical protein | Y-196 | XP_657113 |
| **EHIYNNIKK** | *Plasmodium falciparum* | hypothetical protein | Y-17 | XP_001351358 |
| **EPIYSTNKK** | *Tetrahymena thermophila* | hypothetical protein | v470 | XP_001019273 |
| **EPIYTATKK** | *Trichomonas Vaginalis* | hypothetical protein | Y-3434 | XP_001324654 |
| **EEIYATIDK** | *Candidatus Kuenenia* | hypothetical protein | Y-331 | CAJ7404 |
| **EPLYAVTIK** | *Leishmania braziliensis* | hypothetical protein | Y-143 | XP_001564708 |
| **ESIYHRKYK** | *Tetrahymena thermophila* | hypothetical protein TTHERM_01044690 | Y-2640 | XP_001030642 |
| **ESIYHRKYK** | *Tetrahymena thermophila* | hypothetical protein TTHERM_01044690 | Y-285 | XP_001030642 |
| **NNIYLNIKK** | *Plasmodium yoelii* | CCAAT-box DNA binding protein subunit B | Y-924 | XP_726604 |
