# Supplementary material for: Effector prediction in host-pathogen interaction based on a Markov model of a ubiquitous EPIYA motif
Source: BMC Genomics. 2010 Dec 1;11(Suppl 3):S1. doi: 10.1186/1471-2164-11-S3-S1 (PMC2999339; doi:10.1186/1471-2164-11-S3-S1)
Supplement: Additional File 5 — This file contains a list of sequences that are similar to the R4 motif in bacteria and protista. [file 1471-2164-11-S3-S1-S5.doc]

Additional File 5: R4-like motif sequences in bacteria and protista

| **R4 motif** | **Species** | **Protein** | **pY position** | **Locus** |
| --- | --- | --- | --- | --- |
| **EPIYATIDD** | *H.pylori* | CagA | Y-972 | NP_207343 |
| **EEIYALVDD** | *Citrobacter freundii* | alpha-galactosidase | Y-431 | BAB20428 |
| **ESIYEEIKD** | *Ehrlichia sp.* | ankyrin-like protein, 160K | Y-456 | T08612 |
| **EPIYAEVYD** | *Wolbachia pipientis* | ankyrin domain protein | Y-193 | AAY54257 |
| **ESIYAEIYD** | *Wolbachia pipientis* | ankyrin domain protein | Y-219 | AAY54257 |
| **EPIYARVDL** | *Wolbachia pipientis* | ankyrin domain protein | Y-277 | AAY54257 |
| **EPIYATICA** | *Methylocella silVestris* | cytochrome c class I | Y-190 | YP_002361423 |
| **EEIYALVDD** | *Salmonella enterica* | alpha-galactosidase | Y-430 | NP_458597 |
| **EPLYASLDD** | *Rhodopseudomonas palustris* | hypothetical protein RPD_2343 | Y-216 | YP_569476 |
| **EPLYTLIAD** | *Sinorhizobium medicae* | hypothetical protein Smed_6306 | Y-47 | YP_001314836 |
| **EDIYAGIEF** | *Wolinella succinogenes* | PutatiVe isocitrate dehydrogenase | Y-174 | NP_907613 |
| **ETIYAALAD** | *Anaplasma marginale* | prolyl-tRNA synthetase | Y-357 | YP_153790 |
| **EDIYENIDE** | *Candidatus Pelagibacter* | segregation and condensation protein b | Y-216 | YP_266386 |
| **EEIYESLDD** | *Neorickettsia sennetsu* | hypothetical protein NSe_0051 | Y-408 | YP_505952 |
| **EPIYETIDP** | *Anaplasma marginale* | hypothetical protein AM470 | Y-248 | YP_153762 |
| **EPLYAMITD** | *Rhodopseudomonas palustris* | Phenylacetic acid degradation-related | Y-46 | YP_569019 |
| **EHIYADIRD** | *Pasteurella multocida* | filamentous hemagglutinin | Y-2550 | AAK61595 |
| **ENLYAEISD** | *Pasteurella multocida* | filamentous hemagglutinin | Y-2651 | AAK61595 |
| **EPVYADLHF** | *Haemophilus ducreyi* | large supernatant protein | Y-3398 | NP_873623 |
| **EPVYADLRF** | *Haemophilus ducreyi* | large supernatant protein | Y-3478 | NP_873623 |
| **EPVYADLHF** | *Haemophilus ducreyi* | large supernatant protein | Y-3717 | NP_873623 |
| **EPVYADLRF** | *Haemophilus ducreyi* | large supernatant protein | Y-3797 | NP_873623 |
| **EPVYADLHF** | *Haemophilus ducreyi* | large supernatant protein | Y-4036 | NP_873623 |
| **EPVYADLRF** | *Haemophilus ducreyi* | large supernatant protein | Y-4116 | NP_873623 |
| **EPIYEEIGG** | *Chlamydophila pneumoniae* | hypothetical protein CPj0472 | Y-346 | NP_300527 |
| **EPIYDEIPW** | *Chlamydophila pneumoniae* | hypothetical protein CPj0472 | Y-681 | NP_300527 |
| **DHIYADIND** | *Haemophilus somnus* | cysteine protease domain, YopT-type | Y-2729 | YP_001784809 |
| **NPIYESADA** | *Pasteurella multocida* | PfhB2 | Y-2319 | NP_244996 |
| **EHLYAELDF** | *Bartonella tribocorum* | hypothetical protein Btr_1705 | Y-148 | YP_001610012 |
| **ESIYAPQDP** | *Bartonella tribocorum* | hypothetical protein Btr_1705 | Y-219 | YP_001610012 |
| **EPIYDEVYD** | *Wolbachia endosymbiont* | ankyrin repeat domain protein, putatiVe | Y-77 | ZP_03334563 |
| **ESIYAEVYD** | *Wolbachia endosymbiont* | ankyrin repeat domain protein, putatiVe | Y-100 | ZP_03334563 |
| **EPIYEDVDD** | *Wolbachia endosymbiont* | ankyrin repeat domain protein, putatiVe | Y-130 | ZP_03334563 |
| **EPIYAKVDL** | *Wolbachia endosymbiont* | ankyrin repeat domain protein, putatiVe | Y-155 | ZP_03334563 |
| **EPIYAAVND** | *Agrobacterium Vitis* | ABC transporter substrate binding protein (dipeptide) | Y-370 | YP_002547203 |
| **ESYYSTIEE** | *Lawsonia intracellularis* | hypothetical protein LI0041 | Y-130 | YP_594419 |
| **EPIYAELDF** | *Lawsonia intracellularis* | hypothetical protein LI0666 | Y-149 | YP_595041 |
| **NPIYATVIG** | *Bradyrhizobium sp.* | Hypothetical protein BRADO3491 | Y-111 | YP_001205499 |
| **NPIYAAVAG** | *Methylobacterium populi* | protein of unknown function DUF161 | Y-122 | YP_001927279 |
| **EPLYAQVVG** | *Proteus mirabilis* | PutA | Y-13 | AAF33248 |
| **ESIYAQVPG** | *Methylocella silvestris* | ABC transporter related | Y-747 | YP_002363223 |
| **NPIYINLEE** | *Paramecium tetraurelia* | hypothetical protein | Y-2601 | XP_001453558 |
| **EPLYAVTIE** | *Leishmania infantum* | hypothetical protein | Y-473 | XP_001468457 |
| **EPLYAVTIE** | *Leishmania infantum* | hypothetical protein | Y-695 | XP_001468457 |
| **EPLYAVTIE** | *Leishmania infantum* | hypothetical protein | Y-917 | XP_001468457 |
| **EPLYAVTLE** | *Leishmania infantum* | hypothetical protein | Y-652 | XP_001468598 |
| **EPLYAVTLE** | *Leishmania infantum* | hypothetical protein | Y-918 | XP_001468598 |
| **EPLYAVTLE** | *Leishmania infantum* | hypothetical protein | Y-1001 | XP_001468598 |
| **EPLYAVTLE** | *Leishmania infantum* | hypothetical protein | Y-1267 | XP_001468598 |
| **EPLYAVTLE** | *Leishmania infantum* | hypothetical protein | Y-1350 | XP_001468598 |
| **EPLYAVTLE** | *Leishmania infantum* | hypothetical protein | Y-1433 | XP_001468598 |
| **EPLYAVTLE** | *Leishmania infantum* | hypothetical protein | Y-1516 | XP_001468598 |
| **EPLYAVTLE** | *Leishmania infantum* | hypothetical protein | Y-1599 | XP_001468598 |
| **EPLYAVTLE** | *Leishmania infantum* | hypothetical protein | Y-1682 | XP_001468598 |
| **EPLYAVTLE** | *Leishmania infantum* | hypothetical protein | Y-1765 | XP_001468598 |
| **EPLYAVTID** | *Leishmania braziliensis* | hypothetical protein | Y-12 | XP_001564708 |
| **EPLYAVTIE** | *Leishmania major* | hypothetical protein | Y-473 | XP_001686159 |
| **EPLYAVTIE** | *Leishmania major* | hypothetical protein | Y-695 | XP_001686159 |
| **EPLYAVTIE** | *Leishmania major* | hypothetical protein | Y-917 | XP_001686159 |
| **EPLYAVTID** | *Leishmania major* | hypothetical protein | Y-668 | XP_001686160 |
| **EPLYAVTID** | *Leishmania major* | hypothetical protein | Y-799 | XP_001686160 |
| **EPLYAVTLN** | *Leishmania major* | hypothetical protein | Y-3002 | XP_001686160 |
| **EPLYAVTLE** | *Leishmania major* | hypothetical protein | Y-652 | XP_001686356 |
| **EPLYAVTLE** | *Leishmania major* | hypothetical protein | Y-735 | XP_001686356 |
| **EPLYAVTLE** | *Leishmania major* | hypothetical protein | Y-818 | XP_001686356 |
| **EPLYAVTLE** | *Leishmania major* | hypothetical protein | Y-901 | XP_001686356 |
| **EPLYAVTLE** | *Leishmania major* | hypothetical protein | Y-984 | XP_001686356 |
| **EPLYAVTLE** | *Leishmania major* | hypothetical protein | Y-1067 | XP_001686356 |
| **EPLYAVTLE** | *Leishmania major* | hypothetical protein | Y-1333 | XP_001686356 |
| **EPLYAVTLE** | *Leishmania major* | hypothetical protein | Y-1416 | XP_001686356 |
| **EPLYAVTLE** | *Leishmania major* | hypothetical protein | Y-1499 | XP_001686356 |
| **EPLYAVTLE** | *Leishmania major* | hypothetical protein | Y-1582 | XP_001686356 |
| **EPLYAVTLE** | *Leishmania major* | hypothetical protein | Y-1665 | XP_001686356 |
| **EPLYAVTLE** | *Leishmania major* | hypothetical protein | Y-1748 | XP_001686356 |
| **EPLYAVTLE** | *Leishmania major* | hypothetical protein | Y-1831 | XP_001686356 |
| **EPLYAVTLE** | *Leishmania major* | hypothetical protein | Y-1914 | XP_001686356 |
| **EPLYAVTLE** | *Leishmania major* | hypothetical protein | Y-1997 | XP_001686356 |
| **EPLYAVTLE** | *Leishmania major* | hypothetical protein | Y-2080 | XP_001686356 |
| **EPLYAVTLE** | *Leishmania major* | hypothetical protein | Y-2163 | XP_001686356 |
| **EPLYAVTLE** | *Leishmania major* | hypothetical protein | Y-2246 | XP_001686356 |
| **EPLYAVTLE** | *Leishmania major* | hypothetical protein | Y-2512 | XP_001686356 |
| **EPLYAVTLE** | *Leishmania major* | hypothetical protein | Y-2595 | XP_001686356 |
| **EPLYAVTLE** | *Leishmania major* | hypothetical protein | Y-2678 | XP_001686356 |
| **EPLYAVTLE** | *Leishmania major* | hypothetical protein | Y-2761 | XP_001686356 |
| **EPLYAVTLE** | *Leishmania major* | hypothetical protein | Y-2844 | XP_001686356 |
| **EPLYAVTLE** | *Leishmania major* | hypothetical protein | Y-3110 | XP_001686356 |
| **EPLYAVTLE** | *Leishmania major* | hypothetical protein | Y-3193 | XP_001686356 |
| **EPLYAVTLE** | *Leishmania major* | hypothetical protein | Y-3276 | XP_001686356 |
| **EPLYAVTLE** | *Leishmania major* | hypothetical protein | Y-3359 | XP_001686356 |
| **EPLYAVTLE** | *Leishmania major* | hypothetical protein | Y-3442 | XP_001686356 |
| **EPLYAVTLE** | *Leishmania major* | hypothetical protein | Y-3708 | XP_001686356 |
| **EPLYASVAE** | *Leishmania major* | dynein heavy chain | Y-3033 | XP_001686494 |
| **EPIYQKLRE** | *Entamoeba dispar* | hypothetical protein | Y-882 | XP_001741812 |
| **EPIYELIKE** | *Entamoeba histolytica* | pumilio family RNA-binding protein | Y-118 | XP_657035 |
