# Supplementary material for: Effector prediction in host-pathogen interaction based on a Markov model of a ubiquitous EPIYA motif
Source: BMC Genomics. 2010 Dec 1;11(Suppl 3):S1. doi: 10.1186/1471-2164-11-S3-S1 (PMC2999339; doi:10.1186/1471-2164-11-S3-S1)
Supplement: Additional File 6 — This file contains a list of sequences that are similar to the Tarp motif in bacteria and protista. [file 1471-2164-11-S3-S1-S6.doc]

Additional File 6: Tarp-like motif sequences in bacteria and protista

| **Tarp motif** | **Species** | **Protein** | **pY position** | **Locus** |
| --- | --- | --- | --- | --- |
| **ENIYENIYE** | *Chlamydia trachomatis* | Tarp | Y-136 | YP_001654788 |
| **ESIYENPVE** | *Campylobacter concisus* | motility accessory factor | Y-73 | ABW74832 |
| **EDIYENIDE** | *Candidatus Pelagibacter* | segregation and condensation protein b | Y-216 | YP_266386 |
| **EQIYENLLE** | *Sulfurimonas denitrificans* | diguanylate cyclase (GGDEF domain) | Y-27 | YP_393783 |
| **ESIYENLSE** | *Campylobacter coli* | GTP-binding protein era | Y-195 | ZP_00366822 |
| **ESIYEANYE** | *Campylobacter upsaliensis* | excinuclease ABC, C subunit | Y-568 | ZP_00370333 |
| **EDIYENIDE** | *Candidatus Pelagibacter* | segregation and condensation protein | Y-216 | ZP_01265257 |
| **EHIYEQIGE** | *Haemophilus somnus* | cysteine protease domain, YopT-type | Y-2358 | YP_001784809 |
| **EDIYAEVLE** | *Oligotropha*  *carboxidoVorans* | transcriptional regulator, TetR family | Y-63 | YP_002289222 |
| **EQIYANHSE** | *Tetrahymena thermophila* | HECT domain and RCC1-like domain-containing protein | Y-1544 | XP_001017227 |
| **ESLYEAKVE** | *Tetrahymena thermophila* | HECT domain and RCC1-like domain-containing protein | Y-1978 | XP_001017227 |
| **ESIYTVTAE** | *Tetrahymena thermophila* | hypothetical protein TTHERM_01044690 | Y-82 | XP_001030642 |
| **ENIYEFISR** | *Trichomonas vaginalis* | hypothetical protein | Y-1118 | XP_001306505 |
| **NNIYHNNYH** | *Plasmodium falciparum* | hypothetical protein | Y-918 | XP_001351358 |
| **KKLYINNYE** | *Plasmodium falciparum* | hypothetical protein | Y-3058 | XP_001351358 |
| **KYINNNIYE** | *Plasmodium falciparum* | hypothetical protein | Y-1385 | XP_001351358 |
| **EHIYENVEE** | *Plasmodium falciparum* | hypothetical protein | Y-205 | XP_001351017 |
| **QNNYENVYN** | *Plasmodium falciparum* | hypothetical protein | Y-196 | XP_001350310 |
| **ENKKENIYD** | *Plasmodium falciparum* | hypothetical protein | Y-1180 | XP_001350310 |
| **KVIYKNIYS** | *Plasmodium falciparum* | conserved Plasmodium protein | Y-1198 | XP_001347469 |
| **KQIYEKEYN** | *Plasmodium falciparum* | conserved Plasmodium protein | Y-1477 | XP_001347469 |
| **ERIYEQIDS** | *Paramecium tetraurelia* | hypothetical protein | Y-2164 | XP_001447846 |
| **EKIYTQAFE** | *Trichomonas vaginalis* | hypothetical protein | Y-212 | XP_001581424 |
| **KTIYDNLFR** | *Plasmodium vivax* | hypothetical protein | Y-6800 | XP_001613734 |
| **EIIYSNIKN** | *Plasmodium vivax* | hypothetical protein | Y-62 | XP_001613734 |
| **ETIYENTVG** | *Plasmodium vivax* | hypothetical protein | Y-199 | XP_001617013 |
| **KTIYFNVYP** | *Entamoeba dispar* | hypothetical protein | Y-98 | XP_001741812 |
| **EPIYELIKE** | *Entamoeba histolytica* | pumilio family RNA-binding protein | Y-118 | XP_657035 |
| **EQIYENLMT** | *Entamoeba histolytica* | hypothetical protein | Y-14 | XP_001913580 |
| **DRLWENIYE** | *Entamoeba histolytica* | hypothetical protein | Y-60 | XP_001913580 |
| **ENIYTNSLE** | *Plasmodium yoelii* | CCAAT-box DNA binding protein subunit B | Y-320 | XP_726604 |
| **ESIYKNKLE** | *Plasmodium falciparum* | Plasmodium exported protein (hyp2) | Y-331 | XP_001347309 |
| **ESIYKNKLK** | *Plasmodium falciparum* | Plasmodium exported protein (hyp2) | Y-359 | XP_001347309 |
| **ESIYKNKLK** | *Plasmodium falciparum* | Plasmodium exported protein (hyp2) | Y-387 | XP_001347309 |
