# Supplementary material for: Effector prediction in host-pathogen interaction based on a Markov model of a ubiquitous EPIYA motif
Source: BMC Genomics. 2010 Dec 1;11(Suppl 3):S1. doi: 10.1186/1471-2164-11-S3-S1 (PMC2999339; doi:10.1186/1471-2164-11-S3-S1)
Supplement: Additional File 7 — This file contains a list of sequences that are similar to the Tir motif in bacteria and protista. [file 1471-2164-11-S3-S1-S7.doc]

Additional File 7: Tir-like motif sequences in bacteria and protista

| **Tir motif** | **Species** | **Protein** | **pY position** | **Locus** |
| --- | --- | --- | --- | --- |
| **EHIYDEVAA** | *Escherichia coli* | Tir | Y-470 | AAC38390 |
| **EDIYESVAA** | *Bradyrhizobium sp.* | PutatiVe Cation-transporting ATPase | Y-756 | YP_001239441 |
| **EKIYDLVAT** | *Rhizobium etli* | putative UDP-glucose 4-epimerase protein | Y-26 | YP_470339 |
| **EPIYTFVAA** | *Rhizobium leguminosarum* | putatiVe transmembrane protein | Y-32 | YP_769499 |
| **EQIYDWFAA** | *Hoeflea phototrophica* | polysaccharide deacetylase | Y-291 | ZP_02166931 |
| **EKIYDEVGG** | *Bradyrhizobium japonicum* | putative alkanal monooxygenase | Y-317 | AAM12348 |
| **EPIYDEVYD** | *Wolbachia endosymbiont* | EsorChan1 | Y-77 | AAP34173 |
| **EEIYDDVAS** | *Bradyrhizobium japonicum* | similar to ketol-acid reductoisomerase | Y-61 | NP_772975 |
| **EQIYDEVRE** | *Campylobacter hominis* | hypothetical protein CHAB381_1184 | Y-276 | YP_001406739 |
| **EPVYDKVSA** | *Haemophilus somnus* | cysteine protease domain, YopT-type | Y-2287 | YP_001784809 |
| **EHIYEQIGE** | *Haemophilus somnus* | cysteine protease domain, YopT-type | Y2498 | YP_001784809 |
| **EPLYDEVVG** | *Brucella melitensis* | proline dehydrogenase | Y-443 | AAL53806 |
| **EPIYEEIGG** | *Chlamydophila pneumoniae* | hypothetical protein CPj0472 | Y-346 | NP_300527 |
| **EPIYDEIPW** | *Chlamydophila pneumoniae* | hypothetical protein CPj0472 | Y-681 | NP_300527 |
| **EHLYAEINE** | *Pasteurella multocida* | filamentous hemagglutinin | Y-2387 | AAK61595 |
| **EPVYDKVSA** | *Haemophilus somnus* | cysteine protease domain, YopT-type | Y-2287 | YP_001784809 |
| **ELIYDAVDP** | *Anaplasma marginale* | hypothetical protein AM470 | Y-328 | YP_153762 |
|  |  |  |  |  |
| **EHLYAEINE** | *Pasteurella multocida* | PfhB2 | Y-2387 | NP_244996 |
| **EHIYTDISD** | *Pasteurella multocida* | PfhB2 | Y-2451 | NP_244996 |
| **EPIYETIDP** | *Anaplasma marginale* | hypothetical protein AMF_343 | Y-248 | YP_002563468 |
| **NPIYAEVTG** | *Lactobacillus johnsonii* | hypothetical protein LJ0484 | Y-1159 | NP_964510 |
| **EHIYETIGG** | *Lawsonia intracellularis* | hypothetical protein LIC053 | Y-317 | YP_595669 |
| **EPIYEEIGF** | *Lawsonia intracellularis* | hypothetical protein LIC053 | Y-335 | YP_595669 |
| **ESIYESVSS** | *Lawsonia intracellularis* | hypothetical protein LI0041 | Y-141 | YP_594419 |
| **EPIYAEIKT** | *Lawsonia intracellularis* | hypothetical protein LI0666 | Y-186 | YP_595041 |
| **ENIYQWVAA** | *Trichomonas vaginalis* | hypothetical protein | Y-1092 | XP_001324654 |
| **EDVYDEIVN** | *Trichomonas vaginalis* | hypothetical protein | Y-2236 | XP_001324654 |
| **EHIYNNIKK** | *Plasmodium falciparum* | hypothetical protein | Y-17 | XP_001351358 |
| **EHIYENVEE** | *Plasmodium falciparum* | hypothetical protein | Y-205 | XP_001351017 |
| **EPIYDEKQN** | *Plasmodium falciparum* | cysteine repeat modular protein 2 homologue | Y-689 | XP_001349085 |
| **EKIYDETTN** | *Plasmodium falciparum* | cysteine repeat modular protein 2 homologue | Y-2533 | XP_001349085 |
| **ENIYDNMNC** | *Plasmodium falciparum* | hypothetical protein | Y-1184 | XP_001350310 |
| **EKIYDDNNK** | *Plasmodium falciparum* | conserVed Plasmodium protein | Y-201 | XP_001347469 |
| **EKIYDDNNK** | *Plasmodium falciparum* | conserved Plasmodium protein | Y-210 | XP_001347469 |
| **EKIYDDNNK** | *Plasmodium falciparum* | conserved Plasmodium protein | Y-219 | XP_001347469 |
| **EKIYDDNNK** | *Plasmodium falciparum* | conserved Plasmodium protein | Y-228 | XP_001347469 |
| **EKIYDDNNK** | *Plasmodium falciparum* | conserved Plasmodium protein | Y-237 | XP_001347469 |
| **EKIYDDNNN** | *Plasmodium falciparum* | conserved Plasmodium protein | Y-246 | XP_001347469 |
| **EPLYDADAA** | *Leishmania braziliensis* | cytochrome C oxidase subunit VI | Y-130 | XP_001564903 |
| **EEIYEEICP** | *Trichomonas vaginalis* | hypothetical protein | Y-1062 | XP_001581424 |
| **EDIYDKINA** | *Plasmodium vivax* | hypothetical protein | Y-785 | XP_001615495 |
| **EHIYTKADA** | *Plasmodium vivax* | hypothetical protein | Y-1887 | XP_001615495 |
| **EYLYDDLLP** | *Plasmodium vivax* | hypothetical protein | Y-2262 | XP_001615495 |
| **EPLYDVDAA** | *Leishmania major* | cytochrome C oxidase subunit VI | Y-130 | XP_001683136 |
| **EVLYDEIAN** | *Leishmania major* | hypothetical protein | Y-690 | XP_001686307 |
| **EPLYASVAE** | *Leishmania major* | dynein heaVy chain | Y-3033 | XP_001686494 |
| **EPLYDDVLS** | *Monosiga brevicollis* | hypothetical protein | Y-2947 | XP_001749232 |
| **EETYEGVAS** | *Monosiga brevicollis* | hypothetical protein | Y-4050 | XP_001749232 |
| **EDTYDTVAE** | *Monosiga brevicollis* | hypothetical protein | Y-4160 | XP_001749232 |
| **EDVYDGVET** | *Monosiga brevicollis* | hypothetical protein | Y-4183 | XP_001749232 |
| **EDTYDTVAE** | *Monosiga brevicollis* | hypothetical protein | Y-4239 | XP_001749232 |
| **EDVYDGVEV** | *Monosiga brevicollis* | hypothetical protein | Y-4262 | XP_001749232 |
| **EDVYDGVEV** | *Monosiga brevicollis* | hypothetical protein | Y-4337 | XP_001749232 |
| **ESIYDVVID** | *Plasmodium yoelii* | hypothetical protein | Y-347 | XP_731086 |
| **NYIYDQSAN** | *Plasmodium yoelii* | hypothetical protein | Y-567 | XP_731086 |
