# Supplementary material for: Effector prediction in host-pathogen interaction based on a Markov model of a ubiquitous EPIYA motif
Source: BMC Genomics. 2010 Dec 1;11(Suppl 3):S1. doi: 10.1186/1471-2164-11-S3-S1 (PMC2999339; doi:10.1186/1471-2164-11-S3-S1)
Supplement: Additional File 8 — This file contains top three subcellular localization prediction results of each predicted effector using gram-negative bacterium or eukaryote as the hosting organism, respectively. The values indicate the confidence of the predictions and “*” represents most likely localization. [file 1471-2164-11-S3-S1-S8.doc]

Additional File 7: Subcellular localization prediction results of predict bacterial effectors

| Species | Effector | Gram-negative | | | Eukaryote | | |
| --- | --- | --- | --- | --- | --- | --- | --- |
|  |  | 1^st^ Prediction | 2^nd^ Prediction | 3^rd^ Prediction | 1^st^ Prediction | 2^nd^ Prediction | 3^rd^ Prediction |
| *Bartonella tribocorum* | BepH (YP_001610013, Y-8) | Cytoplasmic  1.669 * | Periplasmic  1.432 * | Extracellular  1.363 * | Nuclear  4.119  * | Extracellular  0.637 | Cytoplasmic  0.115 |
| *Lawsonia intracellularis* | hypothetical protein LI0666 (YP_595041) | Extracellular  2.545 * | Periplasmic  1.335 | OuterMembrane  1.335 | Nuclear  3.154  * | Mitochondrial  0.943 | Cytoplasmic  0.485 |
| *Ehrlichia sp.* | ankyrin (T08612) | Periplasmic  2.003 * | OuterMembrane  1.214 | Extracellular  1.131 | Nuclear  1.419 * | Cytoplasmic  1.373 * | Mitochondrial  0.941 |
| *Wolbachia* | ankyrin (AAY54257) | Extracellular  2.511 * | OuterMembrane  1.723 | Periplasmic  0.42 | Nuclear  3.542 * | Cytoplasmic  0.566 | Mitochondrial  0.248 |
|  | Hypothetical protein WD0942 (NP_966676) | Extracellular  1.709 * | OuterMembrane  1.471 * | Periplasmic  1.275 * | Nuclear  4.265 * | Mitochondrial  0.439 | Cytoplasmic  0.192 |
|  | EsorChan1 (AAP34173) | Cytoplasmic  3.122 * | Periplasmic  0.727 | Extracellular  0.585 | Nuclear  2.181 * | Cytoplasmic  1.470 | Extracellular  0.646 |
| *Pasterurella multocida* | filamentous hemagglutinin (AAK61595) | OuterMembrane  2.344 * | Extracellular  1.723 * | InnerMembrane  0.594 | Nuclear  2.622 * | Cytoplasmic  0.996 | PlasmaMembrane  0.479 |
|  | PfhB2 (NP_244996) | OuterMembrane  2.182 * | Extracellular  1.632 * | InnerMembrane  0.6 | Nuclear  2.374 * | Cytoplasmic  0.990 | PlasmaMembrane  0.482 |
| *Haemophilus ducreyi* | Large supernatant protein2 (NP_873623) | Extracellular  2.318 * | OuterMembrane  1.802 * | InnerMembrane  0.593 | Nuclear  2.638 * | Cytoplasmic  0.550 | PlasmaMembrane  0.484 |
| *Haemophilus somnus* | Cysteine protease domain YopT-type (YP_001784809) | Extracellular  2.245 * | OuterMembrane  1.844 * | InnerMembrane  0.593 | Nuclear  2.551 * | Cytoplasmic  0.944 | PlasmaMembrane  0.482 |
| *Chlamydophila pneumonia* | Hypothetical protein CPj0472 (NP_300527) | OuterMembrane  2.415 * | Extracellular  1.224 | Periplasmic  0.577 | Nuclear  3.099 * | Cytoplasmic  0.949 | PlasmaMembrane  0.286 |
